# Platelet‐Rich Plasma Vs Autologous Blood Vs Corticosteroid Injections in the Treatment of Lateral Epicondylitis: A Systematic Review, Pairwise and Network Meta‐Analysis of Randomized Controlled Trials

**DOI:** 10.1002/pmrj.12287

**Published:** 2020-01-13

**Authors:** Siqi Tang, Xiaoshuai Wang, Peihui Wu, Peiqi Wu, Jiaming Yang, Zefeng Du, Shaoyu Liu, Fuxin Wei

**Affiliations:** ^1^ The Eight Year Program, Zhongshan School of Medicine, Sun Yat‐sen University Guangzhou China; ^2^ Department of Orthopedics Seventh Affiliated Hospital of Sun Yat‐sen University Shenzhen China; ^3^ Department of Joint Surgery First Affiliated Hospital of Sun Yat‐sen University Guangzhou China; ^4^ The Five‐Year Program, School of Medicine, Sun Yat‐sen University Guangzhou China; ^5^ The Five‐Year Program, Zhongshan School of Medicine, Sun Yat‐sen University Guangzhou China

## Abstract

**Objective:**

To compare the effectiveness of platelet‐rich plasma (PRP), autologous blood (AB), and corticosteroid injections in patients with lateral epicondylitis.

**Type of Study:**

Network meta‐analysis.

**Literature Survey:**

Randomized controlled trials (RCTs) that compared any two forms of injections among PRP, AB, and corticosteroid for the treatment of lateral epicondylitis were searched from inception to 30 November 2018, on PubMed, Embase, and Cochrane library.

**Methodology:**

Two researchers independently selected and assessed the quality of RCTs with the Cochrane Risk of Bias Tool. All relevant data from the included studies were extracted and heterogeneity was checked by Cochran's Q test and inconsistency statistic (I^2^). Publication bias was evaluated by constructing contour‐enhanced funnel plots. Stata 15 software was applied for pairwise meta‐analysis and network meta‐analysis. To explore the efficacy between different follow‐up periods, we considered the duration within 2 months to be short term, whereas 2 months or more was considered long term.

**Synthesis:**

Twenty RCTs (n = 1271) were included in this network meta‐analysis. According to ranking probabilities, corticosteroid ranked first for visual analog score (VAS) (surface under the cumulative ranking [SUCRA] = 90.7), modified Nirschl score (82.9), maximum grip strength (69.5), modified Mayo score (MMS) (77.9), and Patient‐Related Tennis Elbow Evaluation (PRTEE) score (93.3) for the short‐term period. For the long‐term period, PRP ranked first for VAS (94.3), pressure pain threshold (99.8), Disabilities of Arm Shoulder and Hand (DASH) score (75.2), MMS (88.2), and the PRTEE score (81.8).

**Conclusion:**

PRP was associated with more improvement in pain intensity and function in the long term than were the comparators. However, in the short term, corticosteroids were associated with the most improvement.

## Introduction

Lateral epicondylitis (LE), also called tennis elbow, is one of the most prevalent upper extremity tendinous disorders. A population study published in 2015 showed that the prevalence of LE in the general population ranged from 1% to 3% and peaked in the fifth decade without gender‐based differences.^1^ The cause of LE remains unclear. However, there is a common agreement that LE might be caused by repetitive strain to the extensor tendon, typically the extensor carpi radialis brevis tendon,^2^, ^3^, ^4^ and by overuse of the wrist. Patients with LE experience pain and lose elbow function.

LE was previously regarded as an inflammatory process; however, recent histopathological studies have demonstrated that the focal site had a paucity of inflammatory cells. Therefore, LE should be considered as tendinosis due to degenerative process of the tendon.^5^ It has been reported that some patients gained benefits from surgical release of the extensor carpi radialis brevis tendon. Percutaneous tenotomy has been considered as one of the effective methods of release.^6^ However, it is limited in clinical practice due to the insufficient and low quality of supporting evidence.^7^ Because of this, most patients prefer to select nonoperative measures, such as activity modification, physical therapy, and injections.^8^ To our knowledge, there is still no consensus on efficacious management or on which therapeutic strategy is the most effective method.^9^, ^10^ Currently, the main nonoperative forms of injection treatment for LE include corticosteroids, autologous blood (AB), and platelet‐rich plasma (PRP). Corticosteroid injection has been reported to be effective in reducing pain and improving function in short‐term follow‐up periods.^11^ Unfortunately, the beneficial effect was diluted with long‐term observation.^12^ AB injections were first used in the management of LE by Edward et al in 2003. It had been demonstrated that AB could trigger an inflammatory reaction around the tendon to promote tissue healing with cellular and humoral mediators.^13^ PRP is collected from the patient's own peripheral blood, which has a high concentration of platelets and platelet‐derived growth factors that augmented the healing process in the tendon.^14^ It has been demontrasted that PRP could enhance tendon regeneration by improving the thickness of the tendon, increasing vascularity and improving tendon morphology.^15^

The aim of our study was to perform a systematic review and network meta‐analysis of randomized controlled trials (RCTs) that compared the clinical effectiveness of corticosteroid, AB, and PRP injections for the management of LE.

## Materials and Methods

### *Search Strategy*

A comprehensive search strategy was conducted for all potentially relevant studies with the use of PubMed, Embase, and Cochrane Library from inception to 30 November 2018. The searches were based on the following keywords: platelet‐rich plasma, corticosteroid, autologous blood, lateral epicondylitis, tennis elbow, and randomized controlled trial. Additional studies were identified by reviewing the reference lists of eligible studies and using the “related articles” features in the electronic database.

### *Inclusion Criteria*

Related studies were included if they matched the following criteria: (1) they were designed as randomized controlled trials; (2) they were in the English language; (3) they compared at least two of the following LE managements – PRP, AB, and corticosteroid; (4) they compared at least one of the following outcomes – visual analog score (VAS), pressure pain threshold (PPT), modified Nirschl score (MNS), Disabilities of Arm Shoulder and Hand (DASH) score, maximum grip strength (MGS), modified Mayo score (MMS), and Patient‐Related Tennis Elbow Evaluation (PRTEE) score; and (5) they reported explicit values of outcomes mentioned previously, including sample capacity, mean, and SD. Studies were excluded if they met exclusion criteria: nonrandomized studies, retrospective studies, reviews, commentaries, animal studies, and unpublished studies.

### *Study Selection*

Study selection was conducted independently by two researchers (Siqi Tang and Peiqi Wu). The disagreements were resolved through discussions with the third researcher (Xiaoshuai Wang).

### *Data Extraction*

Two investigators (Siqi Tang and Peiqi Wu) designed standardized data extraction forms and independently extracted all relevant data from the included studies. If there was missing information, we contacted the corresponding authors of the included trials to request their data. The data collection included (1) general information about the studies (including author, publication year, country, study design, time frame); (2) characteristics of participants (including the number of patients, intervention, mean age, gender, dominant side, and duration); and (3) characteristics of outcomes (including the number of participants, mean, and SD of VAS, PPT, MNS, MGS, DASH score, MMS, and PRTEE score). Discrepancies in data extraction were resolved through discussions with a third investigator (Xiaoshuai Wang).

### *Risk of Bias Assessment*

The Cochrane Risk of Bias Tool of RevMan (Review Manager, V.5.3) was used to assess the qualities of the included studies. A value of “high,” “low,” or “unclear” was assigned based on the following domains: sequence generation, allocation concealment, blinding (participant, personnel, and outcome assessors), incomplete outcome data, selective outcome reporting, and other sources of bias.^16^

### *Outcomes*

The outcomes of interest were pain intensity, strength, and function. The pain score measurement involved VAS, MNS, and PPT. VAS is a single‐item scale, ranging from 0 (no pain) to 100 (worst pain).^17^ MNS assesses pain intensity by level of activity and its scores range from 0 (no pain with exercise) to 4 (severe pain with normal activities).^18^ PPT is measured using algometry and a higher threshold value indicates better pain relief.^19^ Strength was evaluated by MGS, which is a quantitative measure specific for tennis elbow and is obtained with the use of a hand‐held dynamometer.^20^ Functional improvement was evaluated based on the DASH score, MMS, and the PRTEE score. The DASH score includes 30 items with total scores ranging from 0 to 100; a higher score on the DASH indicates worse disability.^21^ MMS ranges from 0 to 100, and a higher score on the MMS represents greater functional improvement.^22^ The PRTEE consists of pain disability and functional disability with a total score ranging from 0 to 100; a higher PRTEE score indicates greater pain and greater dysfunction.^23^

### *Follow‐Up Duration*

There was only one article reporting outcome results 1 year after the treatment. In addition, a large percentage of studies had several follow‐up periods, mainly within 2 months and from 2 months to 1 year. Therefore, the outcome results of different follow‐up duration from one study could be generally divided into two groups, within 2 months (ie, less than 2 months) and 2 months or more. The outcome results of follow‐up within 2 months were derived from the data of the first visit in 2 months after treatment. The outcome results of the 2 months or more follow‐up were collected from the data of the final visit 2 months or more after treatment.

### *Statistical Analysis*

Meta‐analyses were performed for direct comparisons of the outcomes measured in each study between two of the following three therapies: PRP, AB, and corticosteroid injections. For continuous outcome data, the unstandardized mean difference (UMD) and its 95% confidence interval (CI) were pooled to estimate the difference between groups. The heterogeneity of studies was checked by Cochran's Q test and I^2^ statistic. If heterogeneity was found as determined by a statistically significant Q‐statistic or by I^2^ > 25%, a random‐effects model was used to pool the data; otherwise, a fixed‐effects model was applied.

The network meta‐analysis was conducted to assess treatment effects between various injection treatments by performing a multivariate random‐effects meta‐analysis (mvmeta command).^24^ A network of three therapies was mapped, and the nodes and edges were weighted by the number of participants and studies for the accordant comparison (Supplement Figure 1). Contribution plots were used to indicate the contributions of each direct comparison in the network meta‐analysis estimates (Supplement Figure 2). The predictive interval was calculated to estimate the relative treatment effects in other populations (Supplement Figure 3). Contour‐enhanced funnel plots were evaluated to check publication bias (Supplement Figure 4).^25^ To facilitate the interpretation of estimated treatment effects, we utilized the SUCRA (surface under the cumulative ranking) method to calculate ranking probability. Meta‐regression was performed to check the sources of heterogeneity (eg, follow‐up time, mean age, dominant side, and duration of disease) if the data were available. All these analyses were performed by using STATA.

## Results

### *Eligible Studies*

**Figure 1:**
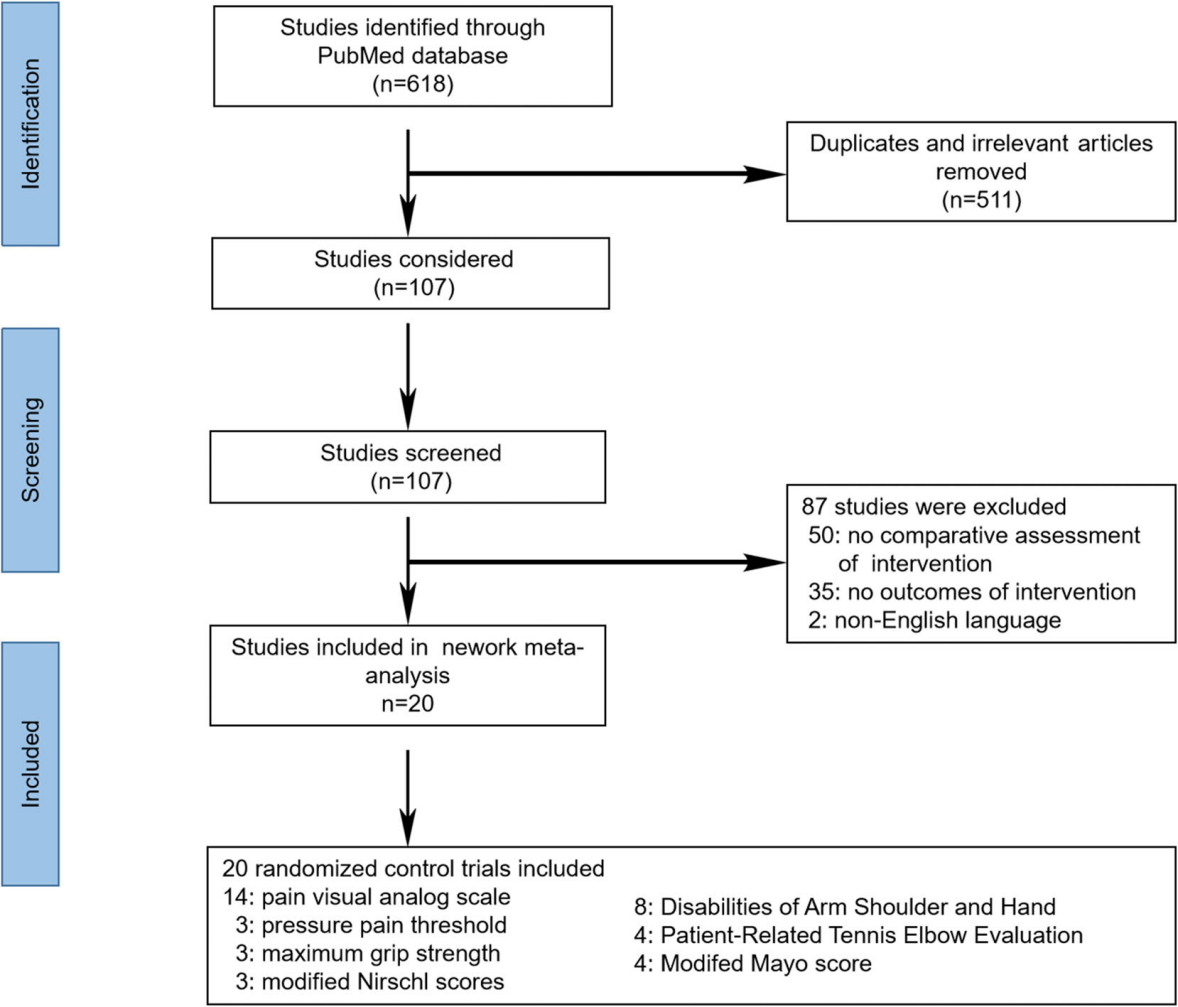
Flowchart of included studies is shown.

Our initial search identified a total of 618 potentially relevant publications. After removing 511 duplicates and irrelevant publications, the titles and abstracts of 107 studies were screened. Full texts were also obtained and scrutinized if necessary. At this stage, 87 studies that did not meet the inclusion criteria were excluded. Twenty eligible RCTs^15^, ^18^, ^20^, ^26^, ^27^, ^28^, ^29^, ^30^, ^31^, ^32^, ^33^, ^34^, ^35^, ^36^, ^37^, ^38^, ^39^, ^40^, ^41^, ^42^ were included in our network meta‐analysis (Figure 1). In 13 PRP trials, the PRP preparation contained a high concentration of leukocytes in three trials^28^, ^37^, ^38^ and was relatively pure with deleted leukocytes in 10 trials.^15^, ^26^, ^30^, ^31^, ^32^, ^33^, ^35^, ^36^, ^40^, ^41^ Most studies^15^, ^20^, ^26^, ^27^, ^28^, ^31^, ^32^, ^34^, ^35^, ^36^, ^37^, ^39^, ^40^, ^41^, ^42^ measured outcomes at more than 2 months; only five studies^18^, ^29^, ^30^, ^33^, ^38^ measured outcomes at 3 weeks to 2 months. The sample size of each treatment group in the trial, average age, and symptom duration varied from 9 to 80, 35.3 to 54 year, and <1 to 35.6 month, respectively. The characteristics of the eligible studies are presented in Table 1.

**Table 1:** Characteristics of the included studies

Author	Study design (evidence level)	Final follow‐up (mo)	Treatment	Preparation of intervention	Cases	Average age (years)	Side (Right/Left)	Mean duration of symptoms (mo)	Outcome measure
Kazemi et al,^18^ 2010; Iran	RCT (2)	2	**CS**	20 mg methylprednisolone + 1 mL lidocaine	30	47.0	NA	1.0‐2.0	VAS, MNS, PPT, MGS, DASH score
								>2.0	
			**AB**	2 mL autologous blood + 1 mL lidocaine	30	47.2	NA	<1.0	
								1.0–2.0	
								>2.0	
Peerbooms et al,^36^ 2010; Netherlands	RCT (1)	12	**CS**	1 mL kenacort 40 mg/mL + 0.5% bupivacaine hydrochloride + epinephrine	51	47.3 ± 7.6	32/19	≥ 6.0	VAS, DASH score
			**PRP**	1 mL PRP + 0.5% bupivacaine hydrochloride + epinephrine	49	46.9 ± 8.4	31/18	≥ 6.0	
Ozturan et al,^34^ 2010; Turkey	RCT (2)	12	**CS**	1 mL methylprednisolone acetate	20	45.8 ± 8.1	NA	9.5 ± 3.1	MGS
			**AB**	2 mL autologous blood	18	44.0 ± 8.5	NA	10.0 ± 2.7	
Creaney et al,^26^ 2011; UK	RCT (1)	6	**PRP**	1.5 mL PRP	63	53.0	NA	≥ 6.0	PRTEE score
			**AB**	1.5 mL autologous blood	48	48.0	NA	≥6.0	
Gosens et al,^28^ 2011; Netherlands	RCT (2)	24	**CS**	1 mL kenacort 40 mg/mL triamcinolon acetonide + 0.5% bupivacaine hydrochloride + epinephrine	49	47.3 ± 7.8	32/17	≥ 6.0	VAS, DASH score
			**PRP**	1 mL PRP + 0.5% bupivacaine hydrochloride + epinephrine	51	46.8 ± 8.5	30/21	≥ 6.0	
Thanasas et al,^40^ 2011; Greece	RCT (1)	6	**AB**	3 mL autologous peripheral whole blood	14	36.6	NA	5.1	VAS
			**PRP**	3 mL autologous PRP	14	35.9	NA	4.7	
Wolf et al,^42^ 2011; United States	RCT (2)	6	**AB**	3 mL autologous blood + lidocaine	10	NA	NA	<6.0	VAS, DASH score
			**CS**	3 mL corticosteroid + lidocaine	9	NA	NA	<6.0	
Dojode^27^ 2012; India	RCT (1)	6	**AB**	2 mL autologous blood + 1 mL 0.5% bupivacaine	30	42.9(22.0‐67.0)*	23/7	9.5(2.0‐54.0)*	VAS, MNS
			**CS**	2 mL local corticosteroid + 1 mL 0.5% bupivacaine	30	42.2(17.0‐62.0)*	23/7	7.7(1.0‐36.0)*	
Omar et al,^30^ 2012; Egypt	RCT (1)	1.5	**CS**	NA	15	37.5 ± 17.5	NA	NA	VAS, DASH score
			**PRP**	NA	15	40.5 ± 15.5	NA	NA	
Jindal et al,^29^ 2013; India	RCT (1)	1.5	**CS**	40 mg methyl prednisolone acetate + 1 mL 2% lignocaine solution	25	37.3 ± 7.5	21/4	4.4 ± 2.4	VAS, MNS
			**AB**	2 mL venous blood + 1 mL 2% lignocaine	25	39.0 ± 6.7	23/2	4.5 ± 1.8	
Singh et al,^39^ 2013; India	RCT (1)	3	**AB**	2 mL venous blood + 1 mL 2% lignocaine	30	35.2 ± 6.8	11/9	7.3 ± 2.5	PRTEE score
			**CS**	40 mg depot methyl prednisolone acetate + 1 mL 2% lignocaine	30	33.0 ± 5.7	9/21	6.9 ± 3.3	
Krogh et al,^31^ 2013; Denmark	RCT (1)	3	**CS**	1 mL 40 mg/mL triamcinolon + 2 mL 10 mg/mL lidocaine	20	43.9 ± 8.7	16/4	35.6 ± 54.1	PRTEE score
			**PRP**	3 mL PRP	20	47.6 ± 7.1	13/7	18.1 ± 36.0	
Raeissadat, Rayegani et al,^37^ 2014; Iran	RCT (1)	12	**PRP**	2 mL autologous PRP	31	43.0 ± 6.0	19/12	>3.0	VAS, PPT, MMS
			**AB**	2 mL autologous peripheral whole blood	30	44.0 ± 7.0	22/8	>3.0	
Raeissadat,Sedighipour et al,^38^ 2014; Iran	RCT (1)	2	**PRP**	2 mL autologous PRP	20	47.2 ± 6.3	11/9	14.5 ± 3.0	VAS, PPT, MMS
			**AB**	2 mL autologous blood	20	45.3 ± 8.7	15/5	14.5 ± 3.0	
Arik et al,^20^ 2014; Turkey	RCT (1)	6	**AB**	2 mL autologous venous blood + 1 mL 2% prilocaine hydrochloride	40	43.7 ± 7.8	9/31	4.3 ± 3.2	VAS, MGS, PRTEE score
			**CS**	40 mg methylprednisolone acetate + 1 mL 2% prilocaine hydrochloride	40	46.7 ± 8.4	14/26	4.5 ± 3.5	
Gautam et al,^15^ 2015; India	RCT (1)	6	**PRP**	2 mL PRP	15	18.0–60.0†	NA	NA	VAS, MGS, DASH score, MMS
			**CS**	2 mL 40 mg/mL methylprednisolone	15	18.0–60.0†	NA	NA	
Khaliq et al,^30^ 2015; Pakistan	RCT (2)	0.75	**CS**	2 mL methylprednisolone acetate + 1 mL 2% xylocaine	51	34.2 ± 10.2	NA	NA	VAS
			**PRP**	3 mL PRP	51	33.6 ± 10.5	NA	NA	
Lebiedzinski et al,^32^ 2015; Poland	RCT (1)	12	**PRP**	NA	53	47.0	NA	NA	DASH score
			**CS**	1 mL betamethasone + 2 mL 1% lignocaine	46	54.0	NA	NA	
Palacio EP et al 35 , 2016; Brazil	RCT (2)	6	**PRP**	3 mL PRP	20	46.6 ± 5.0	NA	NA	DASH score, PRTEE score
			**CS**	3 mL dexamethasoneacetate	20	46.2 ± 5.2	NA	NA	
Varshney A, et al 41, 2017; India	RCT (1)	6	**CS**	80 mg methyl prednisolone + 1 mL lignocaine	50	NA	NA	NA	VAS
			**PRP**	2 mL PRP + 1 mL lignocaine	33	NA	NA	NA	

* Mean (range).

† Range; RCT = randomized controlled trial; CS = corticosteroid; AB = autologous blood; PRP = platelet‐rich plasma; VAS = visual analog score; PPT = pressure pain threshold; MNS = modified Nirschl score; DASH = Disabilities of Arm Shoulder and Hand; MGS = maximum grip strength; MMS = modified Mayo score; PRTEE = Patient‐Related Tennis Elbow Evaluation; NA = not available.

### *Risk of Bias Assessment*

**Figure 2:**
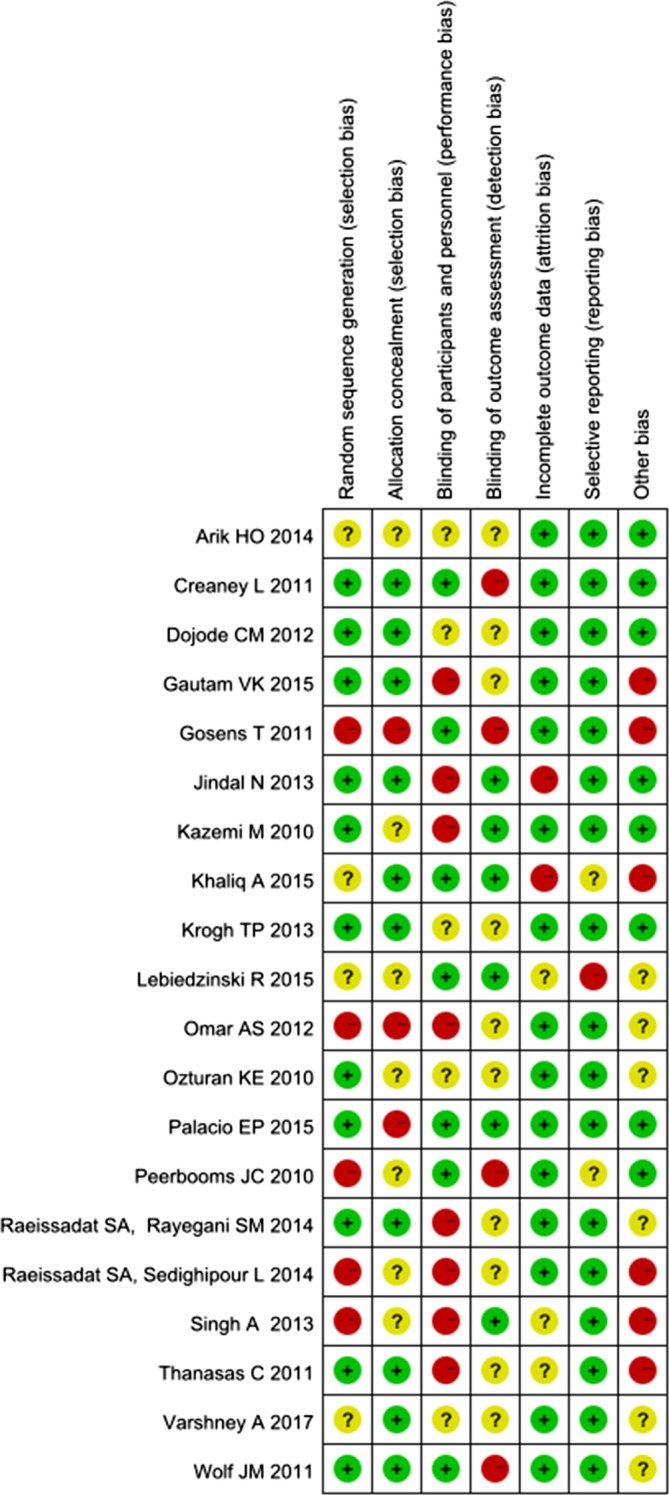
The risk of bias graph is shown.

**Figure 3:**
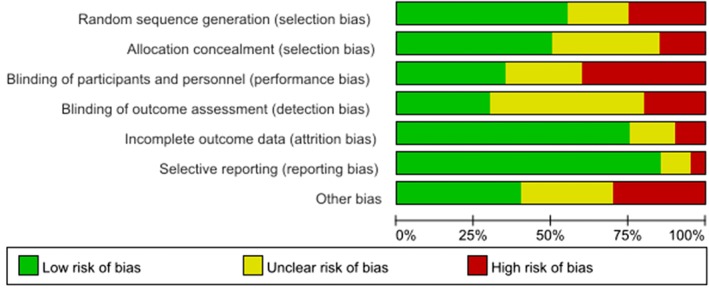
The risk of bias summary is shown.

The risk of bias summary and graph are shown in Figure 2 and Figure 3. Eleven of the included studies generated a low risk of bias in random sequence, and the proper allocation concealment was reported in 10 of these 11 studies. Because these were clinical trials, the implementation of blinding strategies seemed difficult. With reference to the blinding of participants and personnel, only seven of the studies were low risk, and eight were rated as high risk. Furthermore, the blinding of the outcome assessment was clearly presented in only 6 of 20 studies. Fifteen trials included an adequate description of incomplete results, earning a low risk of attrition bias. Only one RCT was rated as high risk in its presentation of reporting bias because it did not define the measurement of pain intensity. Overall, two trials had a low risk of bias, and the remaining 18 trials had an unclear or high risk of bias.

## Results of Pairwise and Network Meta‐Analysis

### *Pain Relief*

In the pairwise meta‐analysis (Table 2), most of the comparisons revealed no significant differences between the groups within the 2‐month follow‐up. At 2 months or more follow‐up, PRP was associated with significantly lower pain scores than corticosteroids (UMD, −2.850; 95% CI, −4.907 to −0.794; *P* = .007) and AB (UMD, −0.747; 95% CI, −1.292 to −0.203; *P* = .007); AB was associated with significantly better changes in pain intensity than corticosteroids (UMD, 1.013; 95% CI, 0.681 to 1.345; *P* < .001); PRP was associated with significantly higher PPT (UMD, 4.400; 95% CI, 1.387 to 7.413; *P* = .004) than AB, and AB was superior to corticosteroids in increasing PPT (UMD, 9.900; 95% CI, 5.593 to 14.207; *P* < .001).

**Table 2:** Summary of direct comparisons according to different interventions

	<2 month				≥ 2 month				
Clinical outcomes	No. of studies	I^2^	No. of participants	UMD (95%CI)	No. of studies	I^2^	No. of participants	UMD (95%CI)	
VAS								
PRP vs CS	6	89.8	214 vs 231	0.847 (−0.345, 2.039)	4	93.3	148 vs 166	−2.850 (−4.907, −0.794) *	
PRP vs AB	3	67.6	65 vs 64	−0.334 (−1.405, 0.737)	3	0	65 vs 64	−0.747 (−1.292, −0.203) *	
AB vs CS	4	97.3	125 vs 125	1.320 (−1.031, 3.670)	4	59.4	109 vs 109	1.654 (0.945, 2.363) *	
MNS									
AB vs CS	3	93.4	85 vs 85	0.602 (−0.745, 1.949)	2	0	55 vs 55	−1.013 (−1.345, −0.681) *	
PPT									
PRP vs AB	2	0	51 vs 50	1.059 (−1.157, 3.276)	1	—	31 vs 30	4.400 (1.387, 7.413) *	
AB vs CS	1	—	30 vs 30	3.800 (0.427, 7.173) *	1	—	30 vs 30	9.900 (5.593, 14.207) *	
MGS									
PRP vs CS	1	—	15 vs 15	−3.000 (−7.160, 1.160)	1	—	15 vs 15	2.600 (−1.946, 7.146)	
AB vs CS	3	87	90 vs 90	−5.126 (−17.207, 6.955)	3	81.4	90 vs 90	10.957 (0.963, 20.952) *	
DASH score									
PRP vs CS	6	81.3	204 vs 196	8.591 (2.452, 14.731)*	5	71.4	188 vs 181	−9.044 (−13.463, −4.625) *	
AB vs CS	2	77.7	39 vs 39	−2.031 (−23.571, 19.509)	2	93.8	39 vs 39	−9.710 (−41.546, 22.127)	
MMS									
PRP vs CS	2	95.6	48 vs 65	−3.193 (−10.983, 4.598)	2	98.8	48 vs 65	20.526 (−1.700, 42.752)	
PRP vs AB	2	0	51 vs 50	0.352 (−5.792, 6.496)	2	0	51 vs 50	6.921 (0.639, 13.203) *	
PRTEE score									
PRP vs CS	2	44.6	40 vs 40	5.033 (2.448, 7.619) *	2	86.2	40 vs 40	−4.472 (−13.655, 4.711)	
PRP vs AB	1	—	80 vs 70	−6.700 (−8.578, −4.822) *	1	—	80 vs 70	−11.000 (−13.401, −8.599) *	
AB vs CS	2	98.5	70 vs 70	18.375 (−7.466, 44.216)	2	85.9	70 vs 70	−9.909 (−19.454, −0.364) *	

* Statistically significant difference (*P* < .05); PRP = platelet‐rich plasma; CS = corticosteroid; AB = autologous blood; VAS = visual analog score; MNS = modified Nirschl score; PPT = pressure pain threshold; MGS = maximum grip strength; DASH = Disabilities of the Arm Shoulder and Hand; MMS = modified Mayo score; PRTEE = Patient‐Rated Tennis Elbow Evaluation; I^2^ = degree of heterogeneity; UMD = unstandardized mean differences; CI = confidence interval.

In terms of the network meta‐analysis (Table 3), there were few significant differences between each of the two treatments within the 2‐month follow‐up. At 2 months or more follow‐up, corticosteroids were associated with significantly lower changes in pain intensity than PRP (MD 2.18; 95% CI, 1.24 to 3.12) and AB (MD 1.60; 95% CI, 0.71 to 2.48) in reducing VAS scores; corticosteroids were significantly inferior to AB in reducing MNS (MD 1.01; 95% CI, 0.68 to 1.35); PRP was associated with significantly higher PPT than AB (MD, 4.40; 95% CI, 1.39 to 7.41) and corticosteroids (MD, 14.30; 95% CI, 9.04 to 19.56); corticosteroids were significantly inferior to AB in improving PPT (MD ‐9.90; 95% CI, −14.21 to −5.59). There was no evidence of significant publication bias as shown by the contour‐enhanced funnel plots (Supplement Figure 4).

**Table 3:** Summary of network meta‐analysis outcomes

Treatment	<2 month		≥2 month	
	Mean difference	95% CI	Mean difference	95% CI
VAS				
PRP vs CS	0.96	(−0.48,2.41)	−2.18	(−3.12,‐1.24)*
PRP vs AB	−0.03	(−1.61,1.54)	−0.58	(−1.58,0.41)
AB vs CS	0.99	(−0.39,2.38)	−1.60	(−2.48,‐0.71)*
MNS				
AB vs CS	0.60	(−0.79,2,00)	−1.01	(−1.35,‐0.68)*
PPT				
PRP vs CS	4.86	(0.82,8.90)*	14.30	(9.04,19.56)*
PRP vs AB	1.06	(−1.16,3.28)	4.40	(1.39,7.41)*
AB vs CS	3.80	(0.34,7.17)*	9.90	(5.59,14.21)*
MGS				
PRP vs CS	−3.01	(−25.11,19.08)	2.62	(−11.68,16.93)
PRP vs AB	2.10	(−23.64,27.84)	−8.16	(−25.16,8.85)
AB vs CS	−5.11	(−18.40,8.18)	10.78	(1.56,20.00)*
DASH score				
PRP vs CS	8.61	(2.01,15.20)*	−11.81	(−23.56,‐0.07)*
PRP vs AB	13.16	(−0.89,27,21)	−1.30	(−23.82,21.22)
AB vs CS	−4.55	(−17.68,8.58)	−10.52	(−29.76,8.73)
MMS				
PRP vs CS	−3.18	(−9.60,3.24)	20.56	(4.39,36.72)*
PRP vs AB	0.17	(−8.61,8.96)	6.77	(−10.30,23.83)
AB vs CS	−3.35	(−14.22,7.52)	13.79	(−9.63,37.22)
PRTEE score				
PRP vs CS	7.72	(−5.49,20.92)	−8.61	(−19.05,1.83)
PRP vs AB	−9.06	(−24.15,6.03)	−2.91	(−14.84,9.03)
AB vs CS	16.78	(3.62,29.93)*	−5.70	(−16.15,4.75)

* Statistically significant difference (*P* < .05); PRP = platelet‐rich plasma; CS = corticosteroid; AB = autologous blood; VAS = visual analog score; MNS = modified Nirschl score; PPT = pressure pain threshold; MGS = maximum grip strength; DASH = Disabilities of the Arm Shoulder and Hand; MMS = modified Mayo score; PRTEE = Patient‐Rated Tennis Elbow Evaluation; I^2^ = degree of heterogeneity; CI = confidence interval.

### *Strength Improvement*

Most of the comparisons revealed no significant differences between the groups in both pairwise meta‐analysis (Table 2) and network meta‐analysis (Table 3). No significant publication bias was detected by the contour‐enhanced funnel plots (Supplement Figure 4).

### *Functional Improvement*

In the pairwise meta‐analysis (Table 2), corticosteroids were associated with lower PRTEE scores (UMD, −5.033; 95% CI, −7.619 to −2.448; *P* < .001) compared with PRP, and PRP was superior to AB in lowering PRTEE scores (UMD, −6.700; 95% CI, −8.578 to −4.822; *P* < .001) within 2 months of follow‐up. At 2 months or more follow‐up, PRP was associated with significantly lower PRTEE scores (UMD, −11.000; 95% CI ‐13.401 to −8.599; *P* < .001) than AB, and AB was significantly superior to corticosteroids in reducing PRTEE scores (UMD, −9.909; 95% CI ‐19.454 to −0.364; *P* = .042).

In terms of the network meta‐analysis (Table 3), there were few significant differences between each of the two treatments, regardless of the follow‐up period. The contour‐enhanced funnel plots for publication bias were not significant (Supplement Figure 4).

### *Ranking – Cumulative Probability*

**Figure 4:**
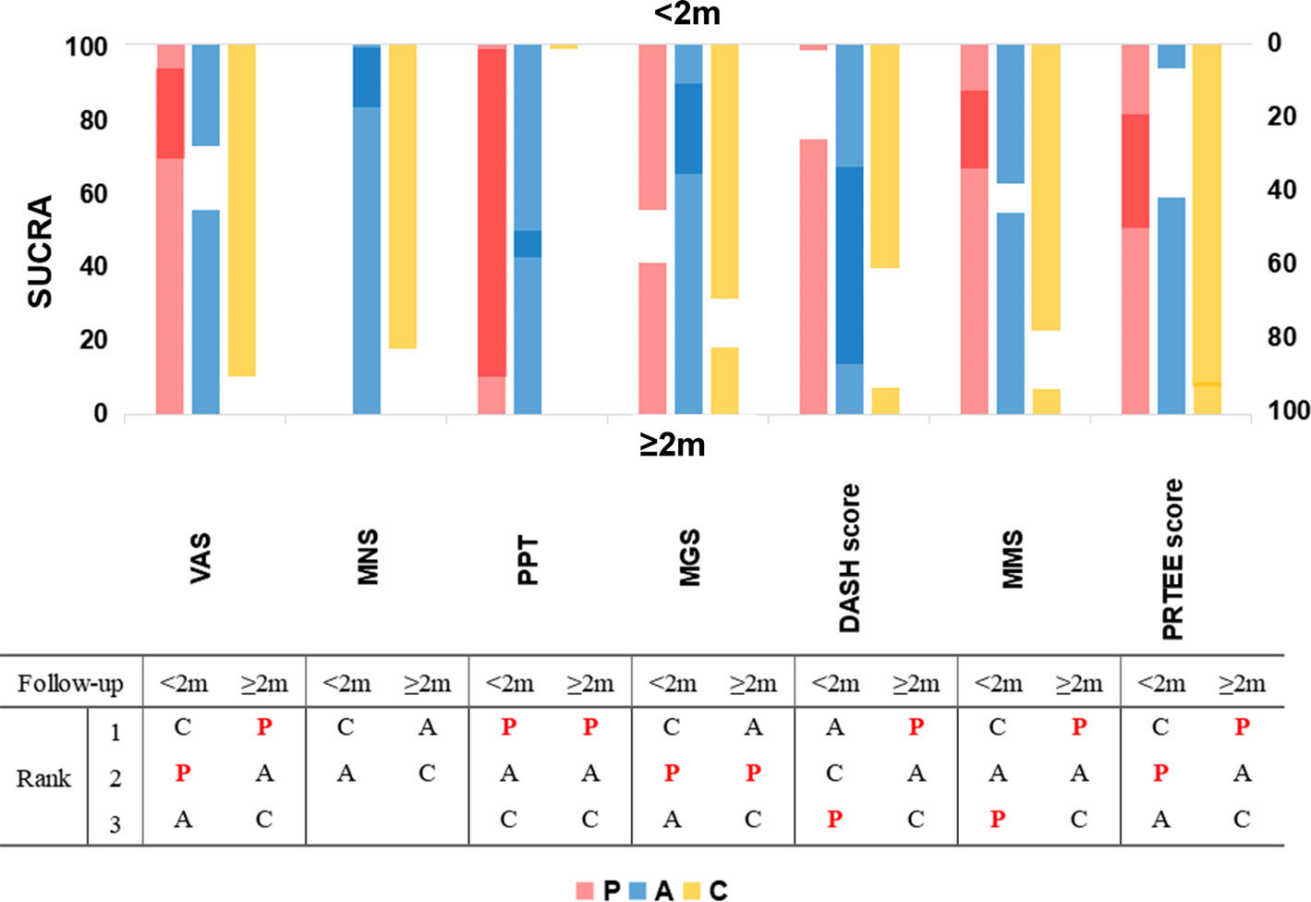
The general rankings of injection treatments in terms of seven different indicators are shown. P = platelet‐rich plasma; A = autologous blood; C = corticosteroid; SUCRA = surface under the cumulative ranking; VAS = visual analog score; MNS = modified Nirschl score; PPT = pressure pain threshold; MGS = maximum grip strength; DASH = Disabilities of the Arm Shoulder and Hand; MMS = modified Mayo score; PRTEE = Patient‐Rated Tennis Elbow Evaluation; <2 m = within 2 months follow‐up; ≥2 m = 2 months or more follow‐up.

Based on the SUCRA method, the probability of each injection being associated with the most improvement for each outcome is presented in Supplement Table 1, and a summarized graph is also provided to facilitate the interpretation (Figure 4). Corticosteroids ranked first in strength improvement and in two of three outcomes of pain reduction as well as functional improvement within 2 months follow‐up. Nevertheless, corticosteroids ranked last in all outcomes at 2 months or more follow‐up. PRP injection ranked first and second with regard to PPT and VAS scores, respectively, within 2 months of follow‐up. In addition, PRP ranked first in two of three pain reduction indicators and in all functional improvement outcomes at 2 months or more follow‐up.

## Discussion

Although there are various noninvasive treatment modalities for LE, to date there has been no consensus concerning the optimal therapeutic approach.^43^ Therefore, we conducted a meta‐analysis and network meta‐analysis to compare three commonly used injections: corticosteroids, PRP, and AB.

There was a previous network meta‐analysis related to this topic.^4^ However, it was noteworthy that 10 additional RCTs had been updated since that report, and most were of high quality. Additionally, our evaluation methods were not completely consistent with the previous study. First, different time points were applied in the meta‐analysis to compare the effects between two follow‐up periods. Second, using the SUCRA method, we ranked probabilities of improvement with each type of injection so that they could be more easily compared. Third, we more comprehensively evaluated pain, strength, and functional outcome measures. In the previous study, the outcomes of interest for evaluating pain were the VAS, DASH, and PRTEE scores. PPT, which was used to assess pain intensity, and the MNS, which were related to the level of activity, were added in our present study.

We adopted the similar follow‐up durations, within 2 months and 2 months or more, in the previous network meta‐study in order to better demonstrate the updated results. Additionally, because the final follow‐up in most of the eligible trials was longer than 2 months and less than 1 year, this demarcation point made the comparison between two follow‐up periods possible. Furthermore, the difference in treatment effectiveness was apparent between two time points in our results, which indicated the rationality of this time node. For easier elaboration, we defined within 2 months as short term, whereas 2 months or more was defined as long term in our study.

Corticosteroids are commonly used to treat LE.^44^ The ranking results indicated that corticosteroids had advantage in the short term, whereas they became the last therapeutic option in the long term. In contrast to other measurements of pain relief in the short term, PPT was not favorably associated with corticosteroids. This might be because there was only one trial that assessed PPT outcomes following corticosteroids. Corticosteroids ranked second in the DASH score, differing slightly from the other two short‐term items related to functional improvement. However, there was no significant difference between corticosteroids and the first ranking treatment; furthermore, the magnitude of the differences between them was small, not to mention the subjectivity of the 30 evaluation items that compose the DASH score. The results in our study were consistent with those in previous network meta‐analyses,^4^ except for two aspects. The outcomes in the former study showed that corticosteroid injections had the worst performance in pain relief within 2 months of follow‐up and had a medium pooled score of functional improvement at 2 months or more follow‐up, which might be due to the small number of included trials. However, our results were supported by the following evidence. In an RCT, Shakeel H et al suggested that the potency behind the earlier pain reduction with a corticosteroid injection could be due to the decrease in inflammation via the arachidonic acid pathway.^45^ However, this effect may not maintain in the long term and may even have adverse effects. An experimental study in rats by Oxlund suggested that the side effects of long‐term local corticosteroid treatment induced a progressive thinning and reduction in collagen in the peroneus longus tendon, which was mainly caused by an inhibition of collagen synthesis.^46^ In a clinical study of lateral epicondylitis, Smidt et al reported that, although clinical results regarding pain, global improvement, and grip strength were favorably associated with corticosteroid injection in the short term, in contrast, there was no significant superiority of corticosteroid treatment in the long term.^47^ Hence, the results of our current study support the preferential use of corticosteroids for a brief duration in the treatment of LE.

PRP is recommended as an ideal autologous blood‐derived product.^48^, ^49^ In the short‐term period, our results suggested that PRP ranked first or second in indexes related to pain. With respect to long‐term results, PRP ranked first in most indicators. Thus, our outcomes highlighted the efficacy of PRP and its correlation with the follow‐up period. In addition to LE, previous evidence has demonstrated the efficacy of PRP in eliminating pain for other tendinopathies, such as gluteal tendinosis, patellar tendinopathy, and rotator cuff injuries. Their observation periods ranged from 6 weeks to more than 1 year.^50^, ^51^, ^52^ Beyond easing pain, clinical evidence of PRP potency in functional improvement was found in treating rotator cuff and refractory Achilles tendinopathies, with follow‐up durations of 6 months and more than 4 years, respectively.^53^, ^54^ The potential biochemical mechanism underlying the temporary pain relief might involve the regulation of inflammation. Platelet‐released IL‐17 significantly recruits neutrophils to resolve inflammation, allowing the reestablishment of normal nociceptive axons and the reduction of their hyperexcitability, thus eliminating neuropathic pain.^55^, ^56^, ^57^ Regarding the persistent curative effect, forming a local environment suitable for regeneration and recovery might also play a key role in pain reduction and functional improvement. PRP likely causes the release of an array of biochemical substances that recruit injured tenocytes and local stem cells, including transforming growth factor (TGF‐β1 and TGF‐β2), platelet‐derived growth factor (PDGF‐AA, PDGF‐AB, and PDGF‐BB), vascular endothelial growth factor (VEGF‐A and VEGF‐C), insulin‐like growth factor 1 (IGF‐1), and epidermal growth factor.^58^, ^59^ Thus, it is noteworthy that PRP injection provides a promising treatment for LE, and more RCTs should focus on confirming its short‐term effect on functional improvement.

In the face of diverse commercially available PRP preparations, the concentration of leukocytes in PRP is a current topic of discussion.^60^ The recent literature suggests that leukocytes exert beneficial effects on antibacterial response and tissue remodeling.^28^, ^61^ In addition, leukocytes are thought to be the main source of growth factors, such as VEGF.^62^, ^63^ A meta‐analysis demonstrated that the application of leukocyte‐rich PRP was preferred for the treatment of chronic tendinopathy.^64^ With regard to LE, there was a relatively large difference between the number of trials evaluating leukocyte‐rich and leukocyte‐poor PRP; there was also diversity in the system of PRP preparation. Therefore, we were unable to conduct an effective comparison and provide a convincing outcome on this issue. To gain a better understanding of this field, more attention should be devoted to the type of PRP that is most suitable for the treatment of LE.

AB injection is another effective treatment in clinical practice. AB mostly ranked second in all aspects at both short‐ and long‐term follow‐ups (Figure 4). This finding contradicted that of a previous network meta‐analysis. The previous study concluded that AB had an advantage in improving function and in pain reduction compared with PRP and corticosteroid injection. This inconsistency may be explained by the relatively small sample size in the earlier study. In addition, recent RCTs have reported inferior outcomes associated with AB. There were several reasons why the AB treatment yielded moderate outcomes. In an animal model, Majewski et al demonstrated the efficacy of AB in promoting tendon healing but did not find improvement in terms of strength.^65^ Furthermore, an ultrasound imaging observational study revealed that injected AB tended to distribute beyond the local area of injection,^66^ which might impair the treatment effect. Therefore, considering that AB injections bring moderate and steady clinical outcomes regardless of the follow‐up duration, we recommend that AB injections should be considered as an alternative treatment for individuals with contraindications to first‐line therapy, or AB should be combined with other methods to optimize its effect.

There were not enough data to conduct a comprehensive analysis of the adverse effects of the different injection therapies; however, the incidence of adverse effects has been reported in a few studies. Thanasas et al^40^ stated that 9 of 14 patients in the PRP‐treated group had local pain and discomfort compared with 4 of 14 patients in the AB‐treated group. Dojode^27^ reported that 2 of 30 patients in the corticosteroid‐treated group had local skin atrophy, whereas no patient had this problem in the AB injection group. Ozturan et al^34^ demonstrated that all patients reported injection pain in the corticosteroid and AB injection groups, whereas more patients in the corticosteroid injection group suffered from delayed relief. Thus, an in‐depth analysis of complications requires more data to support these observations, which are still preliminary.

There are several limitations to this study. First, the injection treatments for LE do not have standardized treatment protocols, which may hinder the comparability of the therapies; therefore, a general agreement on treatment schedules and dosages needs to be investigated in the future. Second, the sources of heterogeneity, such as age and gender, cannot be explored due to insufficient data. Third, the outcome results at 2 months were combined with those at longer term follow‐up. However, only one article reported outcome results 1 year after the treatment. We previously tried to divide the follow‐up time into “short” (ie, less than 2 months), “intermediate” (ie, 2 months to less than 6 months), and “long” (ie, 6 months or more) term. However, no significant change was obtained when an extra time point was added between 2 months and 1 year. The SUCRA results estimated from “intermediate” and “long” term were basically consistent with the results of 2 months or more follow‐up in this study, respectively. The only difference was the rank of autologous blood injection in MMS and PRTEE index during “intermediate” follow‐up. For the “intermediate” term, there was no statistical significance between autologous blood injection and the other two treatments for MMS and PRTEE results, and there were only four articles involving these two indexes, which provided insufficient evidence. Therefore, we think it is difficult to report the short‐, intermediate‐, and long‐term results comprehensively and we would like to apply the follow‐up durations that were reported previously. In the future, more RCTs reporting the outcome results 1 year after treatment are needed to further compare the efficacy among these three therapies in a longer period of follow‐up.

## Conclusion

Among the injection treatments used for lateral epicondylitis, PRP was associated with more improvement in pain intensity and function in the long term than were the comparators. However, in the short term, corticosteroids were associated with the most improvement.

## Author Contributions

All the authors have accepted responsibility for the full contents of this submitted manuscript and approved the submission. Fuxin Wei and Shaoyu Liu conceived and designed the study. Xiaoshuai Wang and Siqi Tang wrote the protocol. Xiaoshuai Wang and Peihui Wu designed and implemented the search strategies. Siqi Tang, Peiqi Wu, and Zefeng Du selected studies, assessed validity, and extracted and analyzed the data. Jiaming Yang verified the data in the tables. All authors were involved in interpreting the results, contributed to the preparation of the full review and its revision, and approved the submission of this manuscript.

## Supporting information


**Appendix S1.** Supporting Information
