# Supplementary material for: Platelet‐Rich Plasma Vs Autologous Blood Vs Corticosteroid Injections in the Treatment of Lateral Epicondylitis: A Systematic Review, Pairwise and Network Meta‐Analysis of Randomized Controlled Trials
Source: PM R. 2020 Jan 13;12(4):397–409. doi: 10.1002/pmrj.12287 (PMC7187193; doi:10.1002/pmrj.12287)
Supplement: Supplementary file 1 — Appendix S1. Supporting Information [file PMRJ-12-397-s001.pdf]

Supplementary materials

Supplement Table 1. Summary of SUCRA outcomes

SUCRA \ Treatment	< 2 months			≥ 2 months		
	PRP	CS	AB	PRP	CS	AB
VAS	31.4	90.7	27.8	94.3	0.0	55.7
MNS	—	82.9	17.1	—	0.0	100.0
PPT	90.7	1.3	58.0	99.8	0.0	50.1
MGS	45.1	69.5	35.4	41.5	18.3	90.2
DASH score	1.7	61.2	87.1	75.2	7.2	67.6
MMS	34.0	77.9	38.0	88.2	6.7	55.1
PRTEE score	50.2	93.3	6.5	81.8	8.9	59.3

SUCRA = Surface Under the Cumulative Ranking Curve; PRP = platelet-rich plasma; CS = corticosteroid; AB = autologous blood; VAS = visual analog score; MNS = modified Nirschl score; PPT = pressure pain threshold; MGS = maximum grip strength; DASH = Disabilities of the Arm Shoulder and Hand; MMS = modified Mayo score; PRTEE = Patient-Rated Tennis Elbow Evaluation.

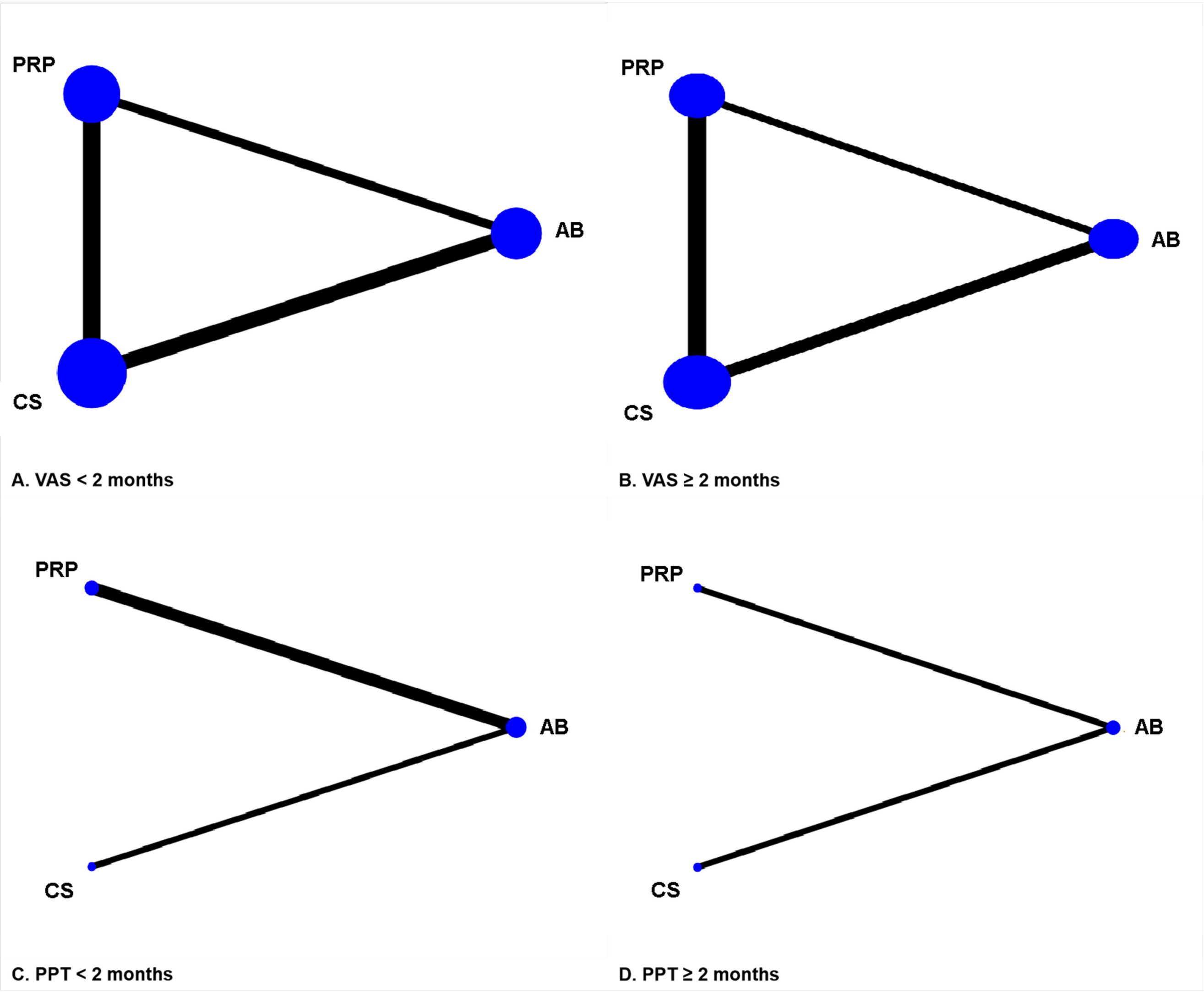
PRP
AB
CS
A. VAS < 2 months
PRP
AB
CS
B. VAS ≥ 2 months
PRP
AB
CS
C. PPT < 2 months
PRP
AB
CS
D. PPT ≥ 2 months

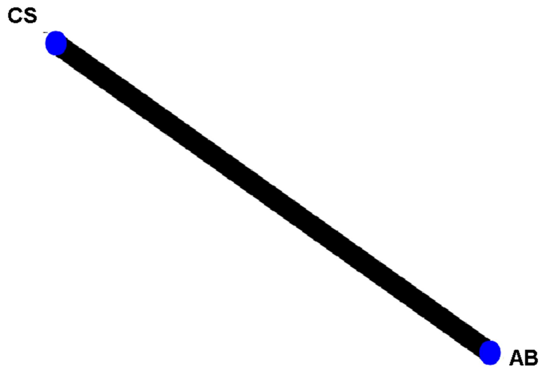

E. MNS < 2 months

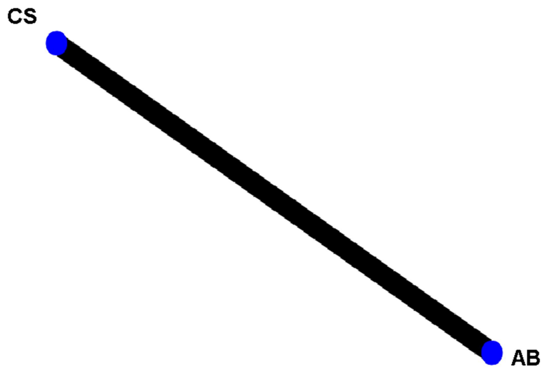

F. MNS ≥ 2 months

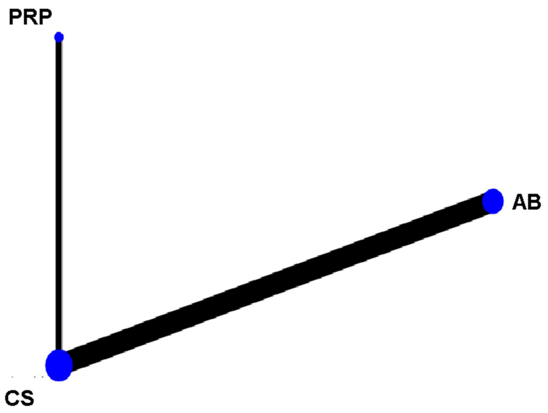

G. MGS < 2 months

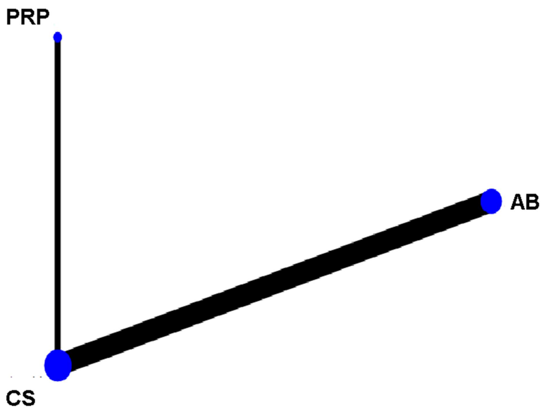

H. MGS ≥ 2 months

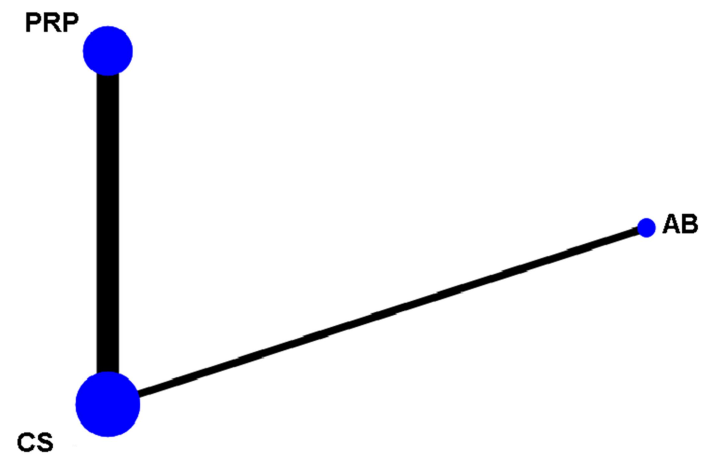

I. DASH < 2 months

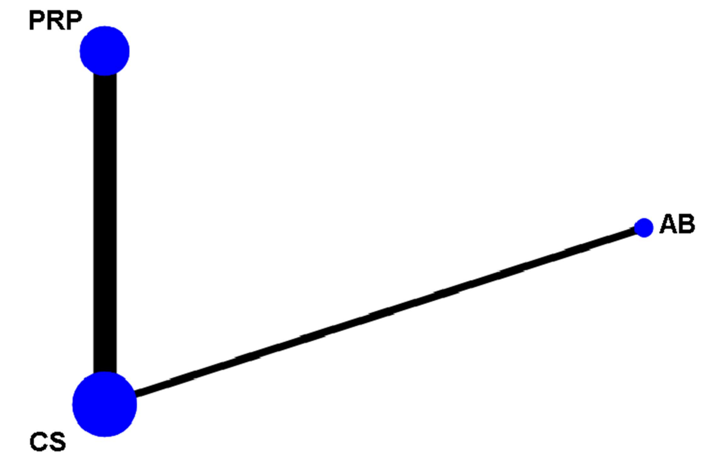

J. DASH ≥ 2 months

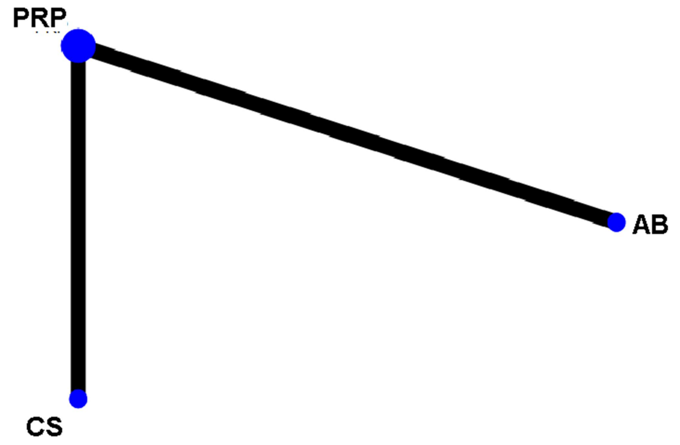

K. MMS < 2 months

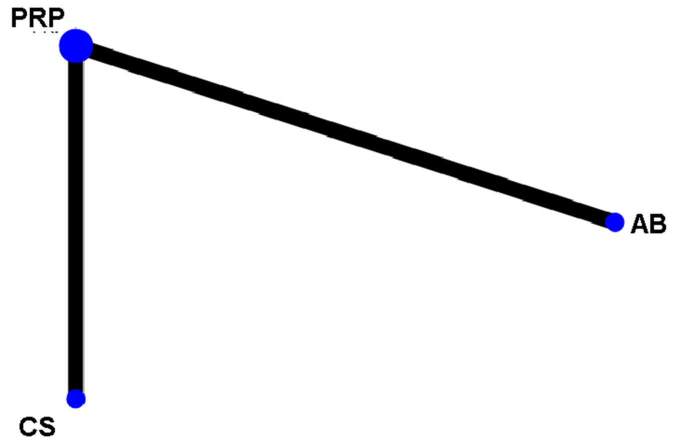

L. MMS ≥ 2 months

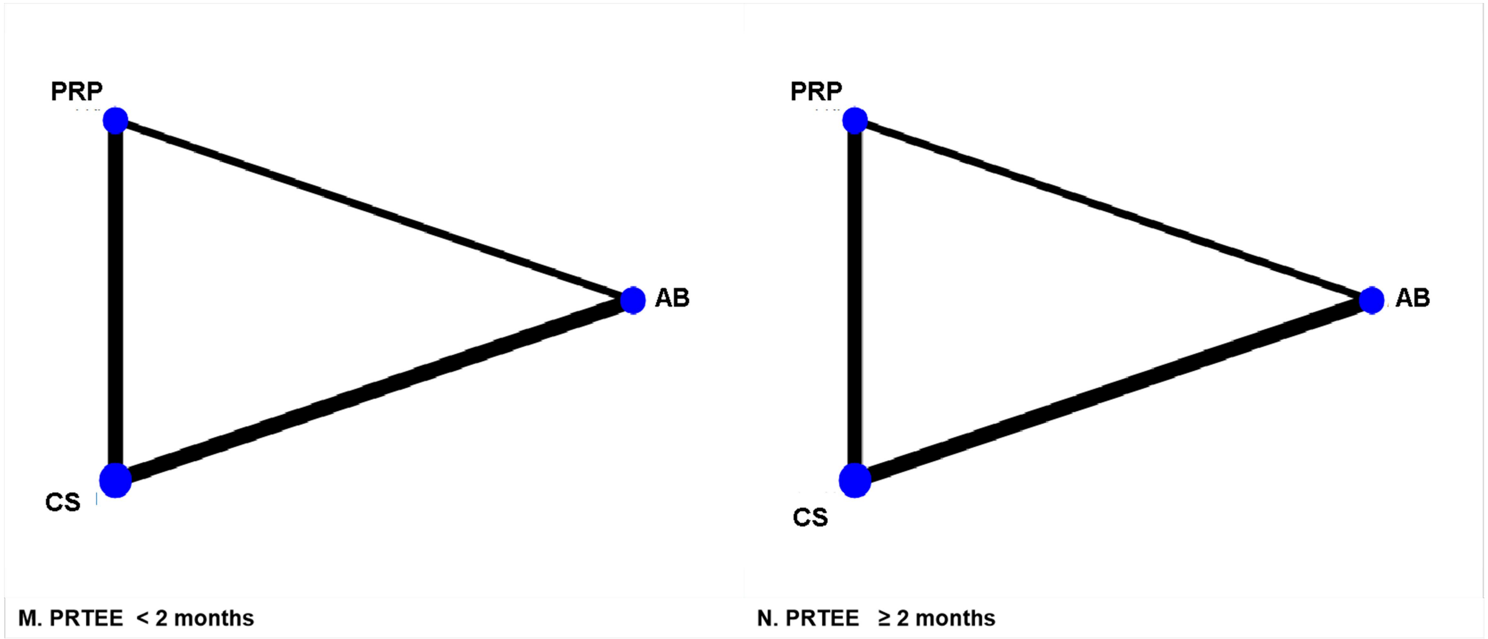

Supplement Fig. 1 The network of injection treatment comparisons is shown. PRP = platelet-rich plasma; AB = autologous blood; CS = corticosteroid; VAS = visual analog score; MNS = modified Nirschl score; PPT = pressure pain threshold; MGS = maximum grip strength; DASH = Disabilities of the Arm Shoulder and Hand; MMS = modified Mayo score; PRTEE = Patient-Rated Tennis Elbow Evaluation.

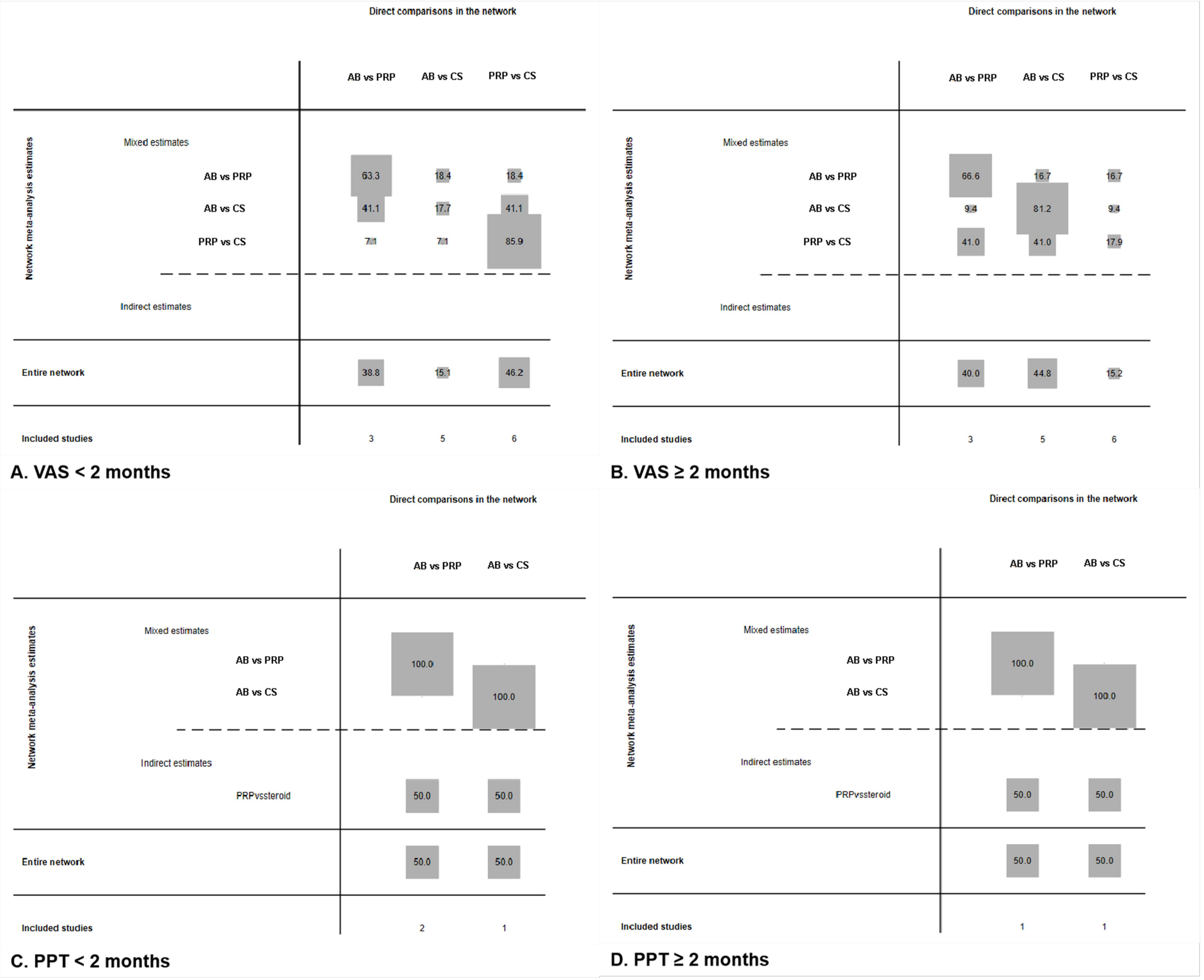
Direct comparisons in the network
AB vs PRP
AB vs CS
PRP vs CS
Network meta-analysis estimates
Mixed estimates
AB vs PRP
63.3
18.4
18.4
AB vs CS
41.1
17.7
41.1
PRP vs CS
7.1
7.1
85.9
Indirect estimates
Entire network
38.8
15.1
46.2
Included studies
3
5
6
A. VAS < 2 months
Direct comparisons in the network
AB vs PRP
AB vs CS
PRP vs CS
Network meta-analysis estimates
Mixed estimates
AB vs PRP
66.6
16.7
16.7
AB vs CS
9.4
81.2
9.4
PRP vs CS
41.0
41.0
17.9
Indirect estimates
Entire network
40.0
44.8
15.2
Included studies
3
5
6
B. VAS ≥ 2 months
Direct comparisons in the network
AB vs PRP
AB vs CS
Network meta-analysis estimates
Mixed estimates
AB vs PRP
100.0
AB vs CS
100.0
Indirect estimates
PRPvssteroid
50.0
50.0
Entire network
50.0
50.0
Included studies
2
1
C. PPT < 2 months
Direct comparisons in the network
AB vs PRP
AB vs CS
Network meta-analysis estimates
Mixed estimates
AB vs PRP
100.0
AB vs CS
100.0
Indirect estimates
PRPvssteroid
50.0
50.0
Entire network
50.0
50.0
Included studies
1
1
D. PPT ≥ 2 months

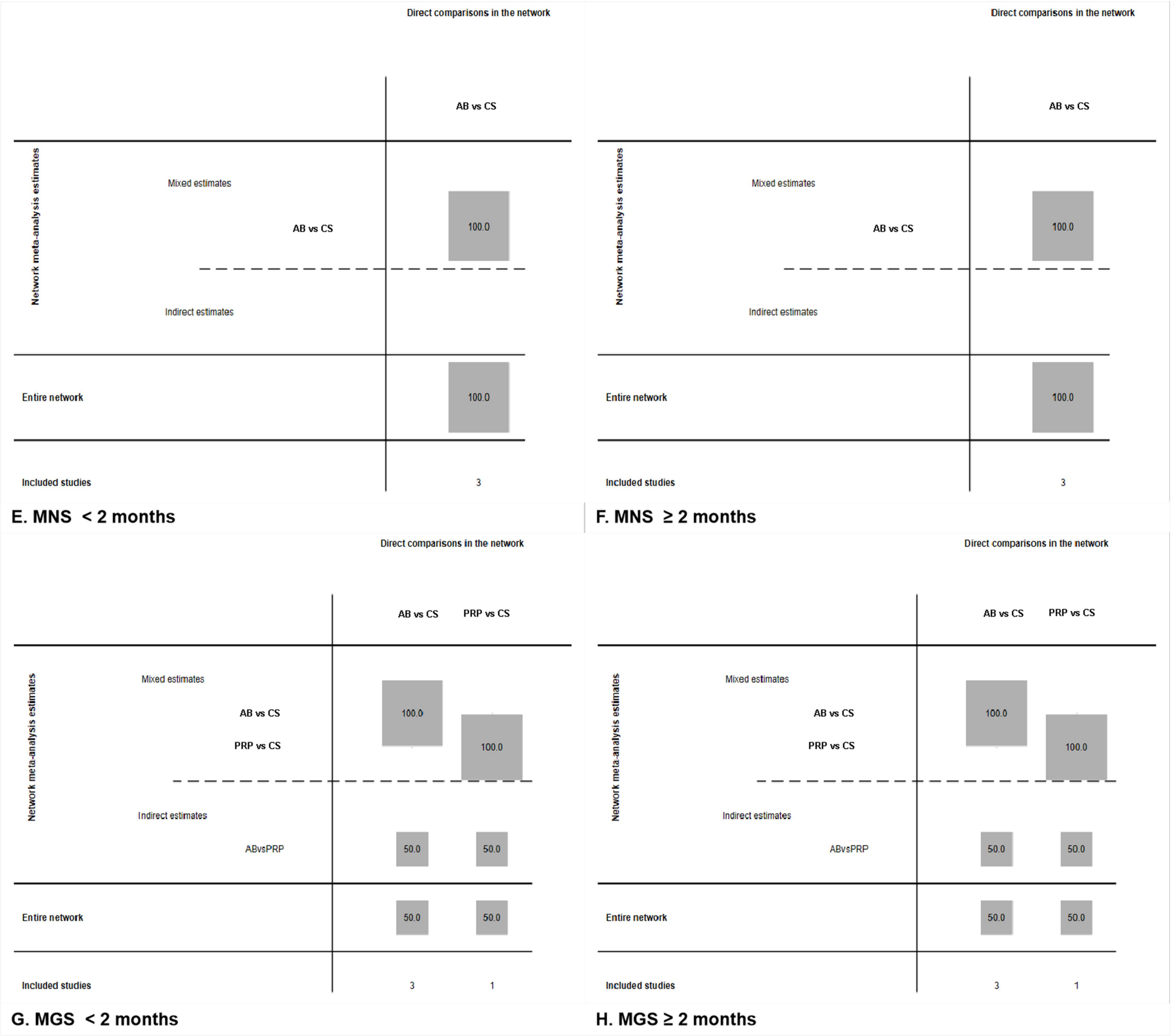
Direct comparisons in the network
AB vs CS
Network meta-analysis estimates
Mixed estimates
AB vs CS
100.0
Indirect estimates
Entire network
100.0
Included studies
3
E. MNS < 2 months
Direct comparisons in the network
AB vs CS
Network meta-analysis estimates
Mixed estimates
AB vs CS
100.0
Indirect estimates
Entire network
100.0
Included studies
3
F. MNS ≥ 2 months
Direct comparisons in the network
AB vs CS
PRP vs CS
Network meta-analysis estimates
Mixed estimates
AB vs CS
100.0
PRP vs CS
100.0
Indirect estimates
ABvsPRP
50.0
50.0
Entire network
50.0
50.0
Included studies
3
1
G. MGS < 2 months
Direct comparisons in the network
AB vs CS
PRP vs CS
Network meta-analysis estimates
Mixed estimates
AB vs CS
100.0
PRP vs CS
100.0
Indirect estimates
ABvsPRP
50.0
50.0
Entire network
50.0
50.0
Included studies
3
1
H. MGS ≥ 2 months

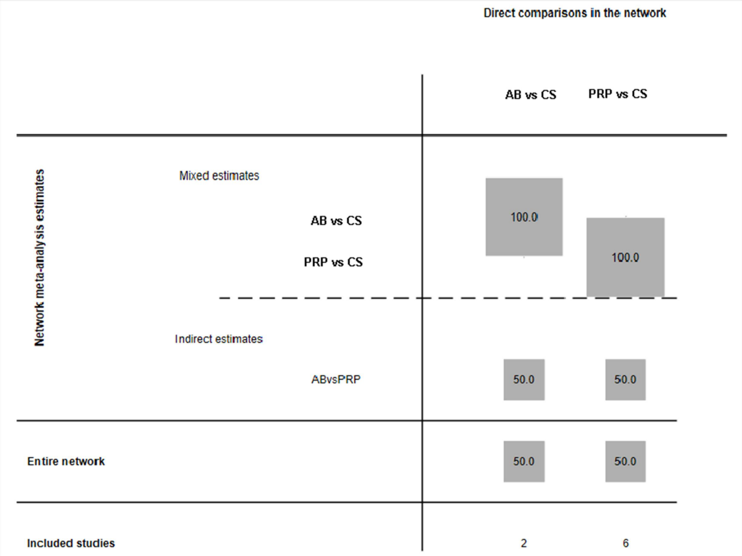

I. DASH < 2 months

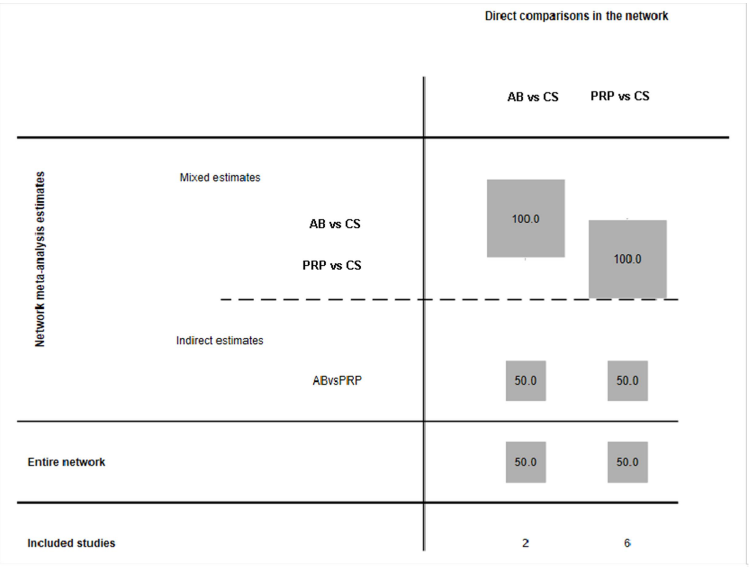

J. DASH ≥ 2 months

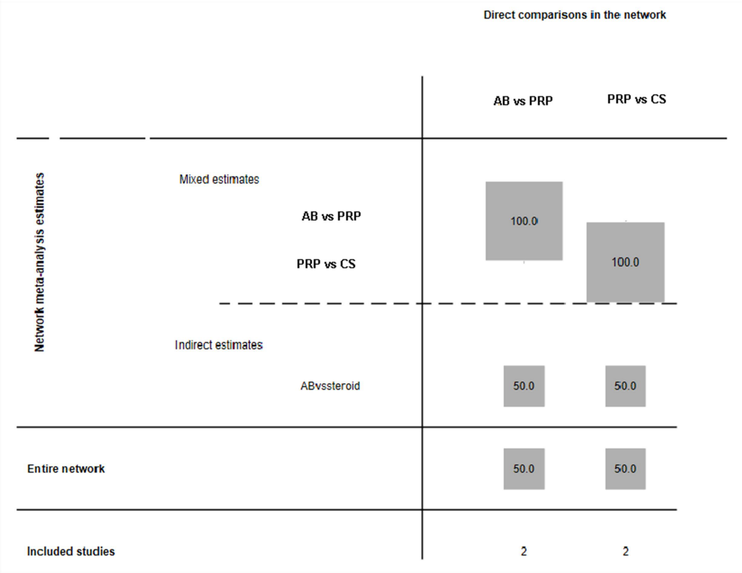

K. MMS < 2 months

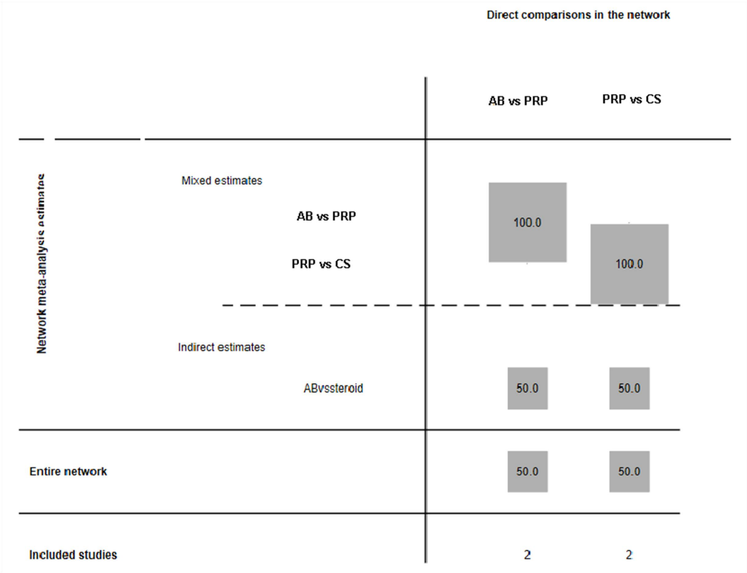

L. MMS ≥ 2 months

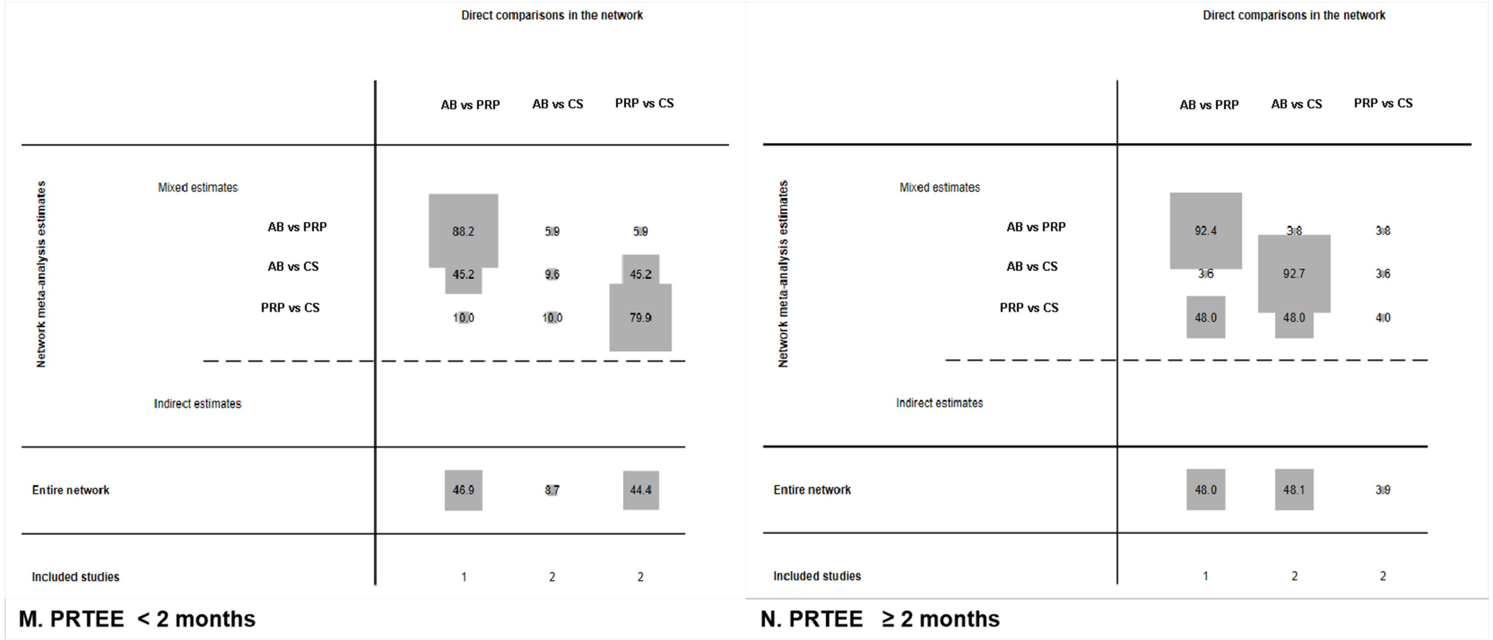

Supplement Fig. 2 The contribution plot is shown. PRP = platelet-rich plasma; AB = autologous blood; CS = corticosteroid; VAS = visual analog score; MNS = modified Nirschl score; PPT = pressure pain threshold; MGS = maximum grip strength; DASH = Disabilities of the Arm Shoulder and Hand; MMS = modified Mayo score; PRTEE = Patient-Rated Tennis Elbow Evaluation.

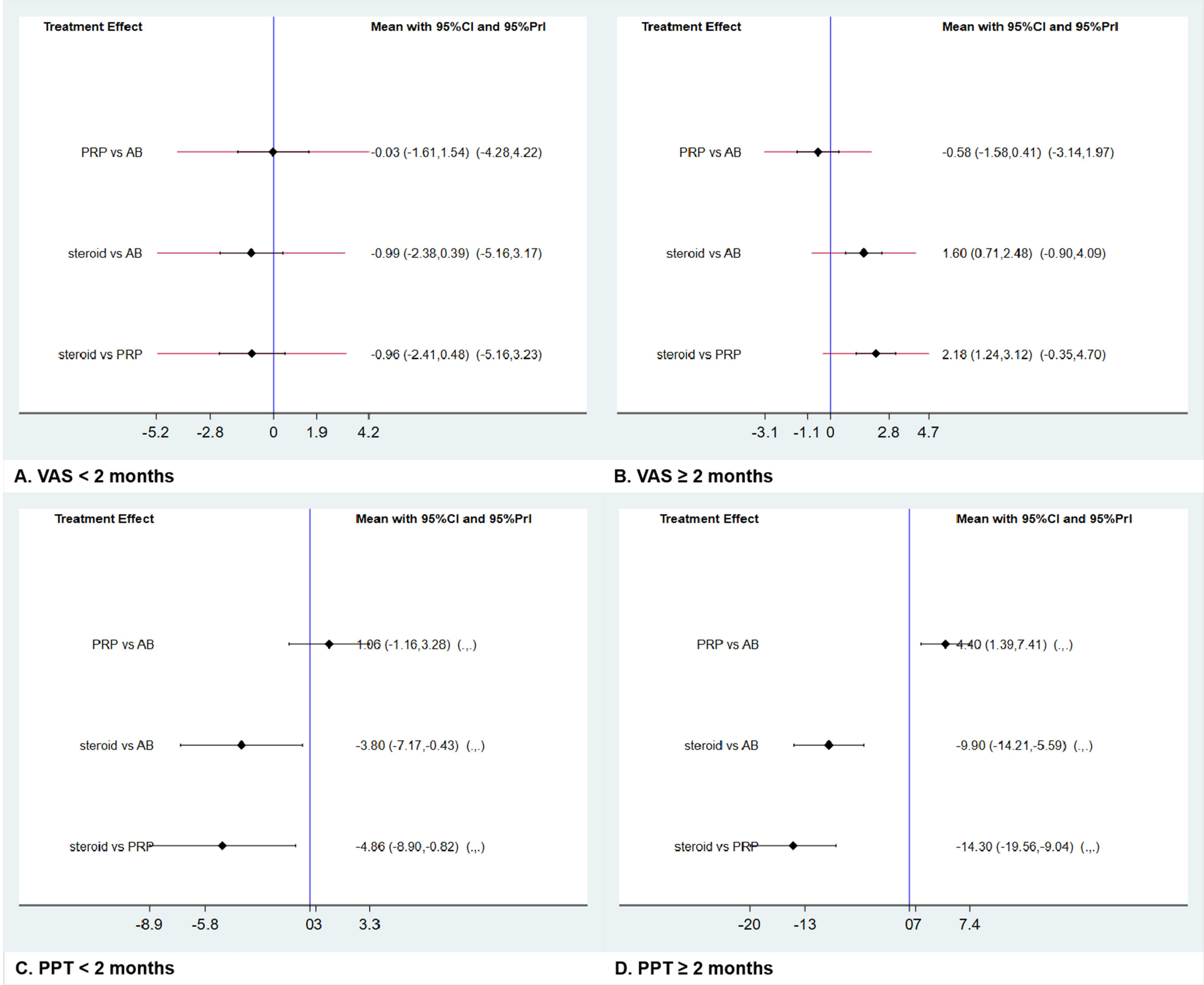

Treatment Effect
Mean with 95%CI and 95%PrI
PRP vs AB
-0.03 (-1.61,1.54) (-4.28,4.22)
steroid vs AB
-0.99 (-2.38,0.39) (-5.16,3.17)
steroid vs PRP
-0.96 (-2.41,0.48) (-5.16,3.23)
-5.2 -2.8 0 1.9 4.2
A. VAS < 2 months
Treatment Effect
Mean with 95%CI and 95%PrI
PRP vs AB
-0.58 (-1.58,0.41) (-3.14,1.97)
steroid vs AB
1.60 (0.71,2.48) (-0.90,4.09)
steroid vs PRP
2.18 (1.24,3.12) (-0.35,4.70)
-3.1 -1.1 0 2.8 4.7
B. VAS ≥ 2 months
Treatment Effect
Mean with 95%CI and 95%PrI
PRP vs AB
1.06 (-1.16,3.28) (.,.)
steroid vs AB
-3.80 (-7.17,-0.43) (.,.)
steroid vs PRP
-4.86 (-8.90,-0.82) (.,.)
-8.9 -5.8 03 3.3
C. PPT < 2 months
Treatment Effect
Mean with 95%CI and 95%PrI
PRP vs AB
4.40 (1.39,7.41) (.,.)
steroid vs AB
-9.90 (-14.21,-5.59) (.,.)
steroid vs PRP
-14.30 (-19.56,-9.04) (.,.)
-20 -13 07 7.4
D. PPT ≥ 2 months

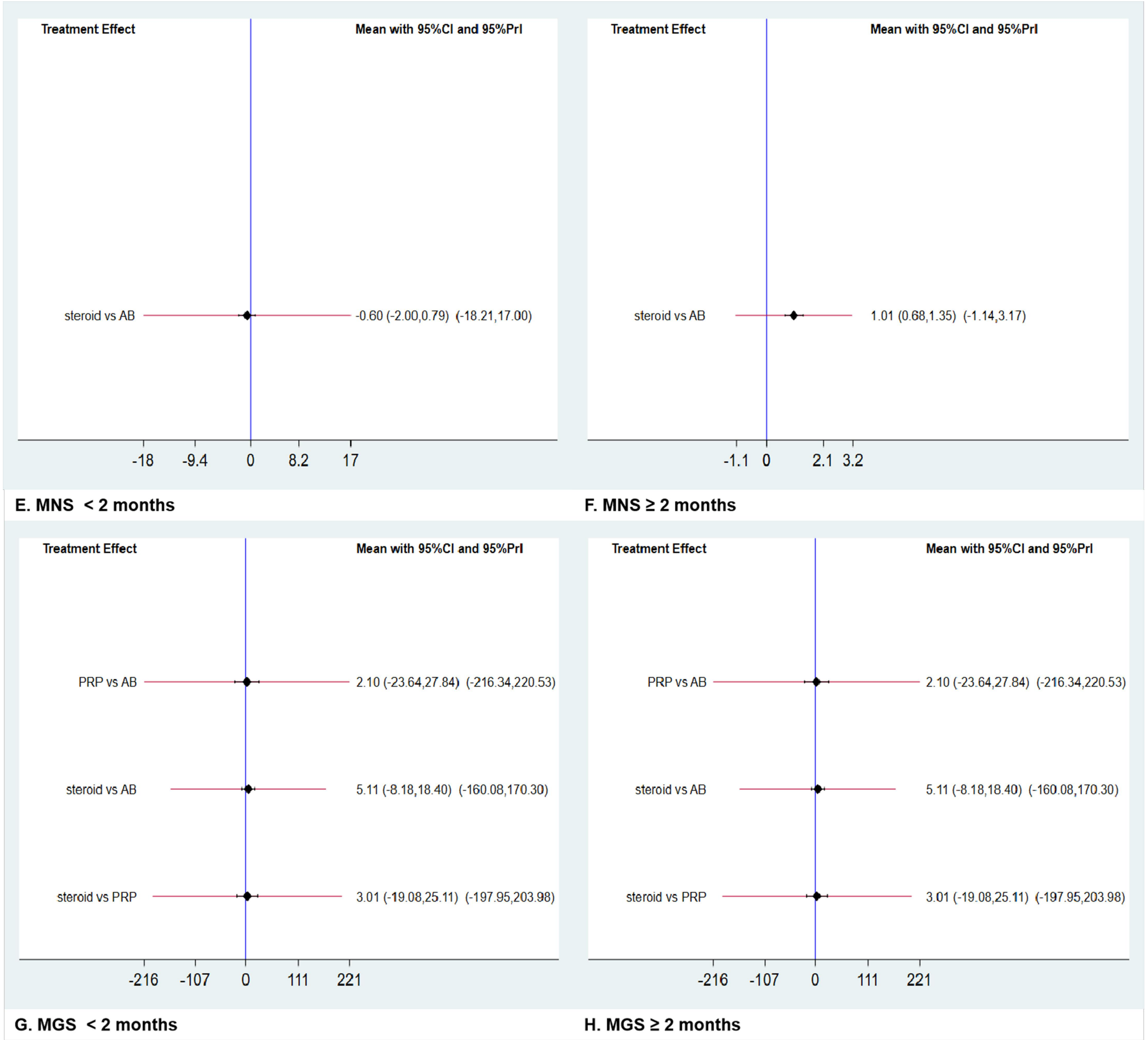

Treatment Effect
Mean with 95%CI and 95%PrI
steroid vs AB
-0.60 (-2.00,0.79) (-18.21,17.00)
-18
-9.4
0
8.2
17
E. MNS < 2 months
Treatment Effect
Mean with 95%CI and 95%PrI
steroid vs AB
1.01 (0.68,1.35) (-1.14,3.17)
-1.1
0
2.1
3.2
F. MNS ≥ 2 months
Treatment Effect
Mean with 95%CI and 95%PrI
PRP vs AB
2.10 (-23.64,27.84) (-216.34,220.53)
steroid vs AB
5.11 (-8.18,18.40) (-160.08,170.30)
steroid vs PRP
3.01 (-19.08,25.11) (-197.95,203.98)
-216
-107
0
111
221
G. MGS < 2 months
Treatment Effect
Mean with 95%CI and 95%PrI
PRP vs AB
2.10 (-23.64,27.84) (-216.34,220.53)
steroid vs AB
5.11 (-8.18,18.40) (-160.08,170.30)
steroid vs PRP
3.01 (-19.08,25.11) (-197.95,203.98)
-216
-107
0
111
221
H. MGS ≥ 2 months

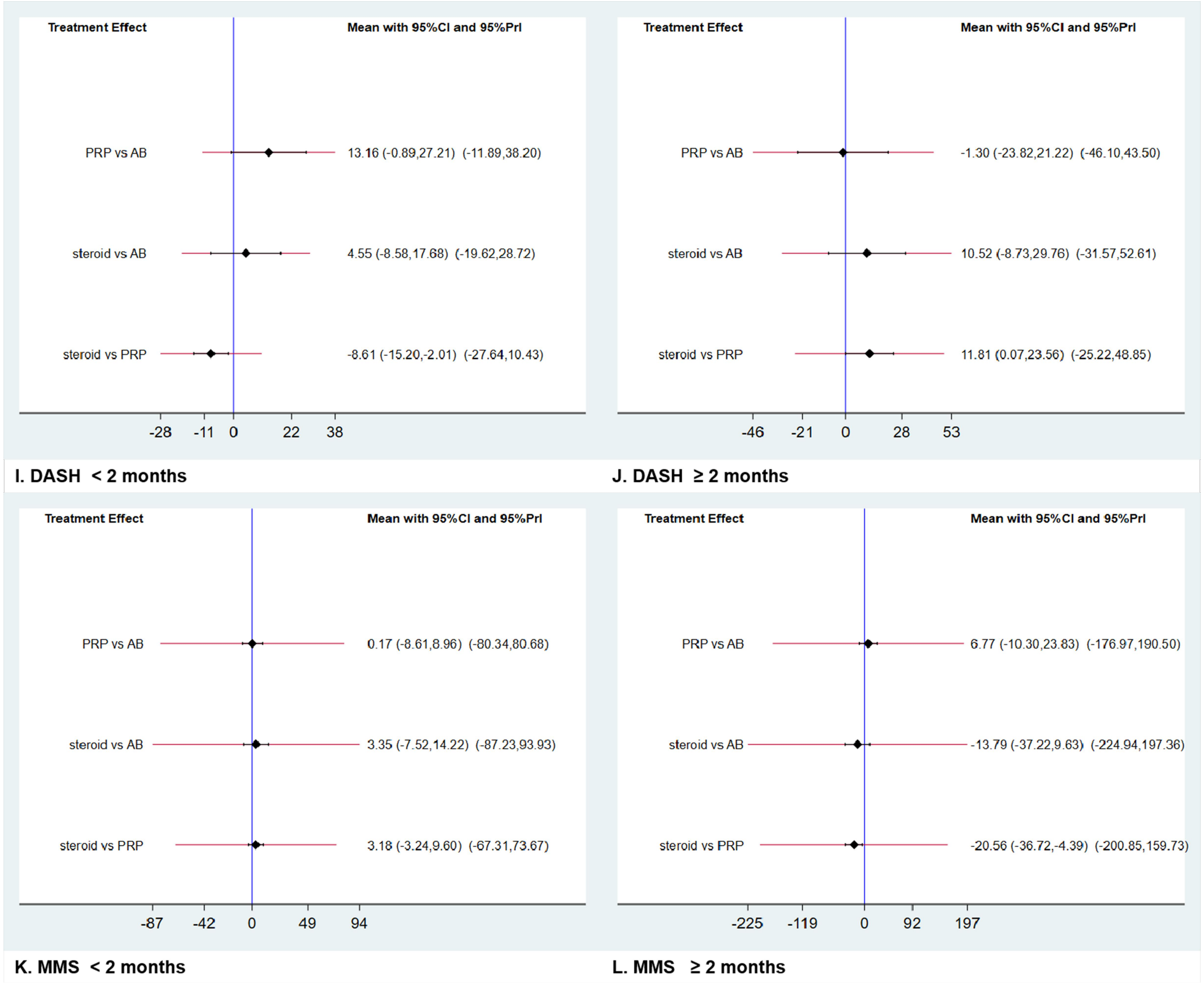

Treatment Effect
Mean with 95%CI and 95%PrI
PRP vs AB
13.16 (-0.89,27.21) (-11.89,38.20)
steroid vs AB
4.55 (-8.58,17.68) (-19.62,28.72)
steroid vs PRP
-8.61 (-15.20,-2.01) (-27.64,10.43)
-28
-11
0
22
38
I. DASH < 2 months
Treatment Effect
Mean with 95%CI and 95%PrI
PRP vs AB
-1.30 (-23.82,21.22) (-46.10,43.50)
steroid vs AB
10.52 (-8.73,29.76) (-31.57,52.61)
steroid vs PRP
11.81 (0.07,23.56) (-25.22,48.85)
-46
-21
0
28
53
J. DASH ≥ 2 months
Treatment Effect
Mean with 95%CI and 95%PrI
PRP vs AB
0.17 (-8.61,8.96) (-80.34,80.68)
steroid vs AB
3.35 (-7.52,14.22) (-87.23,93.93)
steroid vs PRP
3.18 (-3.24,9.60) (-67.31,73.67)
-87
-42
0
49
94
K. MMS < 2 months
Treatment Effect
Mean with 95%CI and 95%PrI
PRP vs AB
6.77 (-10.30,23.83) (-176.97,190.50)
steroid vs AB
-13.79 (-37.22,9.63) (-224.94,197.36)
steroid vs PRP
-20.56 (-36.72,-4.39) (-200.85,159.73)
-225
-119
0
92
197
L. MMS ≥ 2 months

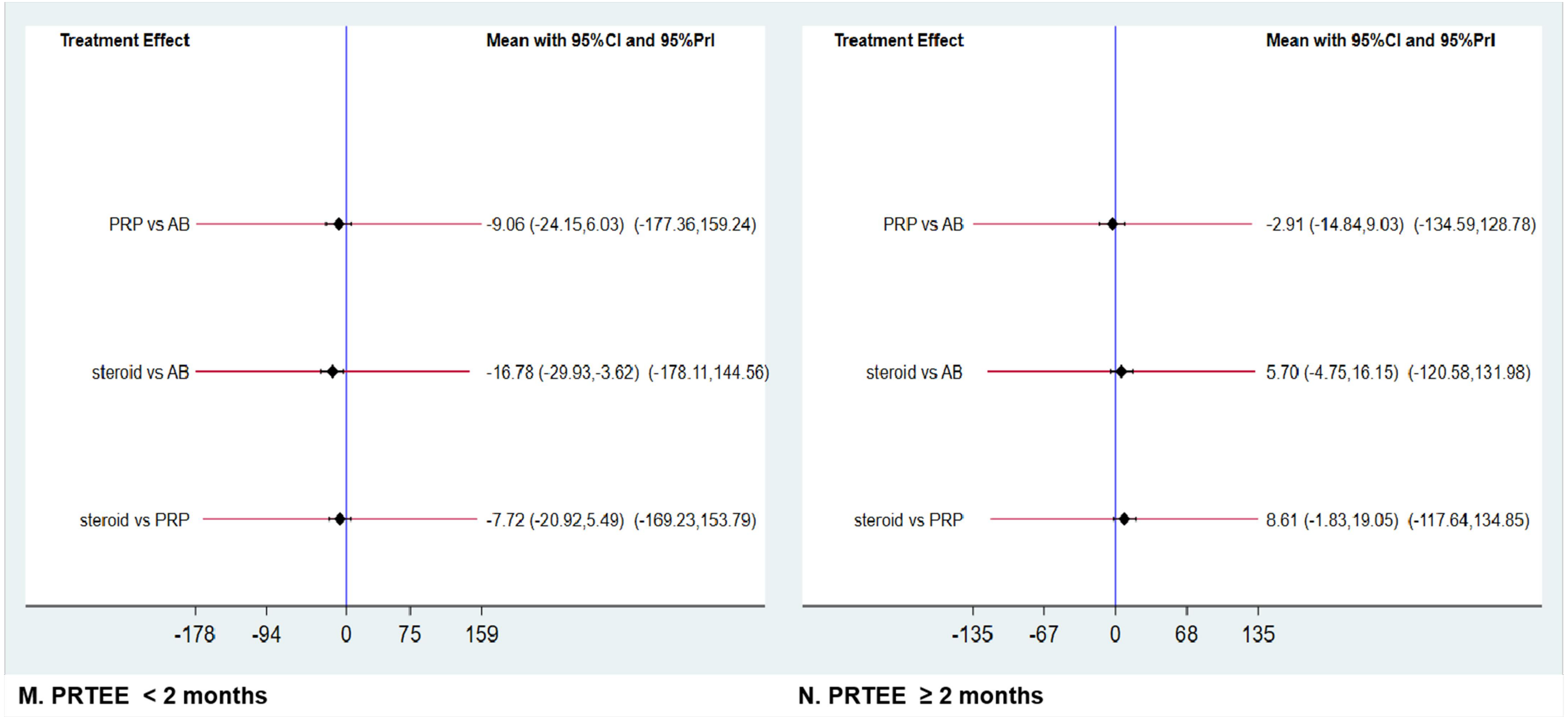

Supplement Fig. 3 The predictive interval plot is shown. PRP = platelet-rich plasma; AB = autologous blood; CS = corticosteroid; VAS = visual analog score; MNS = modified Nirschl score; PPT = pressure pain threshold; MGS = maximum grip strength; DASH = Disabilities of the Arm Shoulder and Hand; MMS = modified Mayo score; PRTEE = Patient-Rated Tennis Elbow Evaluation.

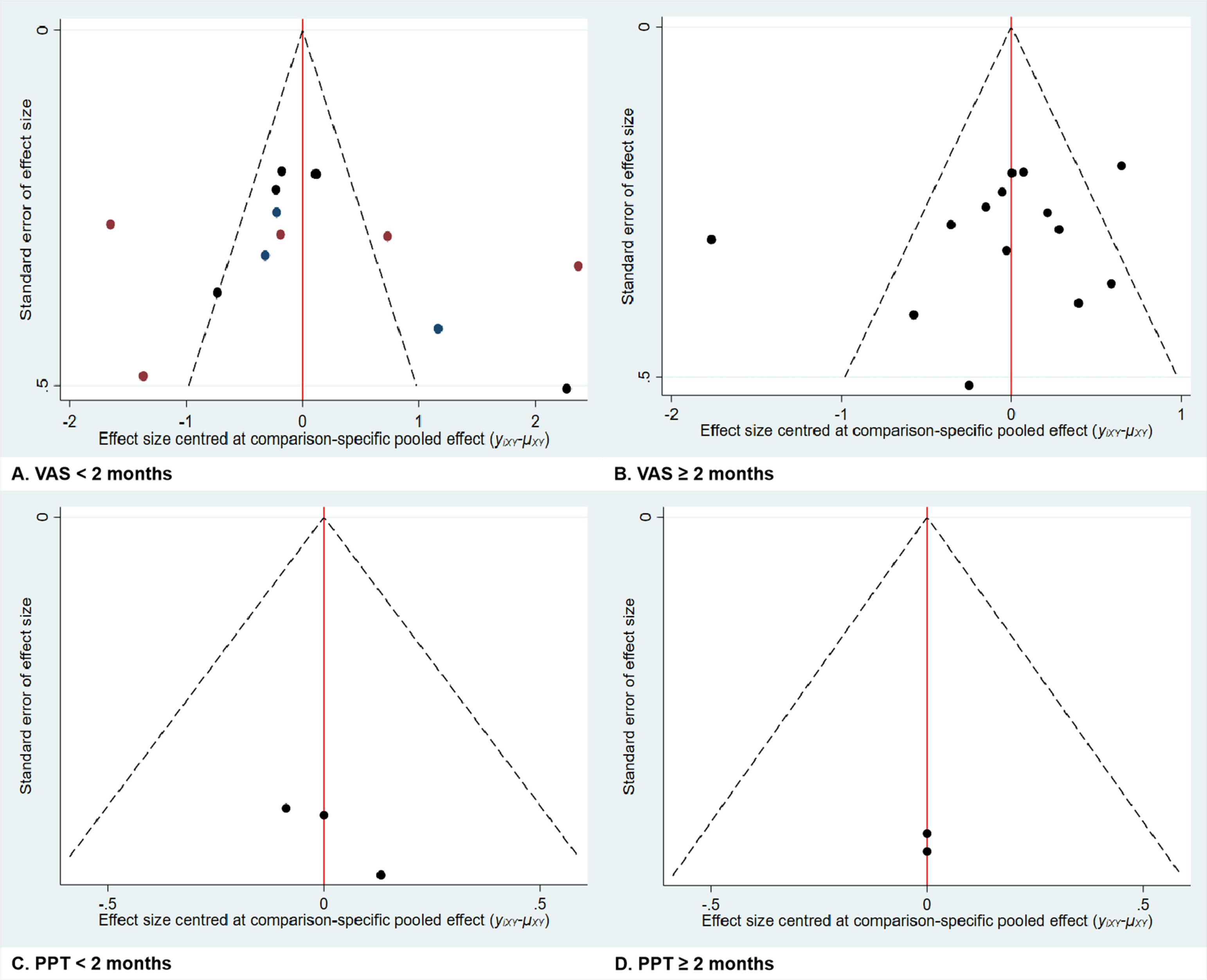
Standard error of effect size
0
.5
-2
-1
0
1
2
Effect size centred at comparison-specific pooled effect (y_{iXY}-μ_{XY})
A. VAS < 2 months
Standard error of effect size
0
.5
-2
-1
0
1
Effect size centred at comparison-specific pooled effect (y_{iXY}-μ_{XY})
B. VAS ≥ 2 months
Standard error of effect size
0
-.5
0
.5
Effect size centred at comparison-specific pooled effect (y_{iXY}-μ_{XY})
C. PPT < 2 months
Standard error of effect size
0
-.5
0
.5
Effect size centred at comparison-specific pooled effect (y_{iXY}-μ_{XY})
D. PPT ≥ 2 months

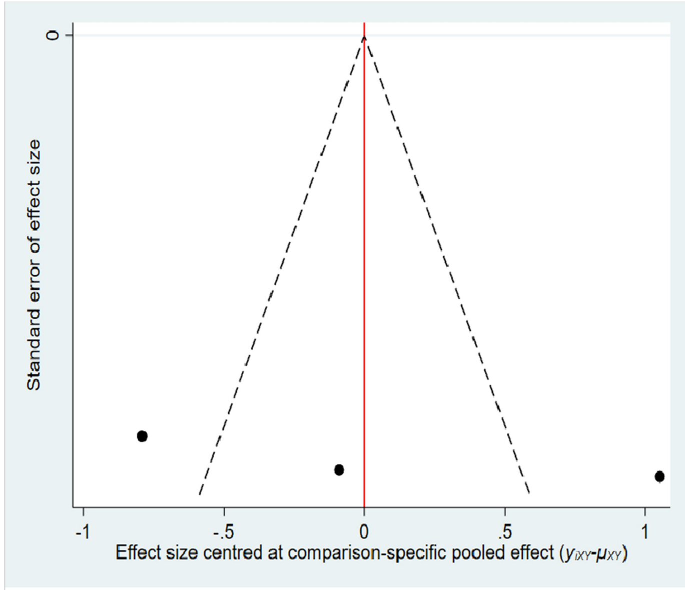

E. MNS < 2 months

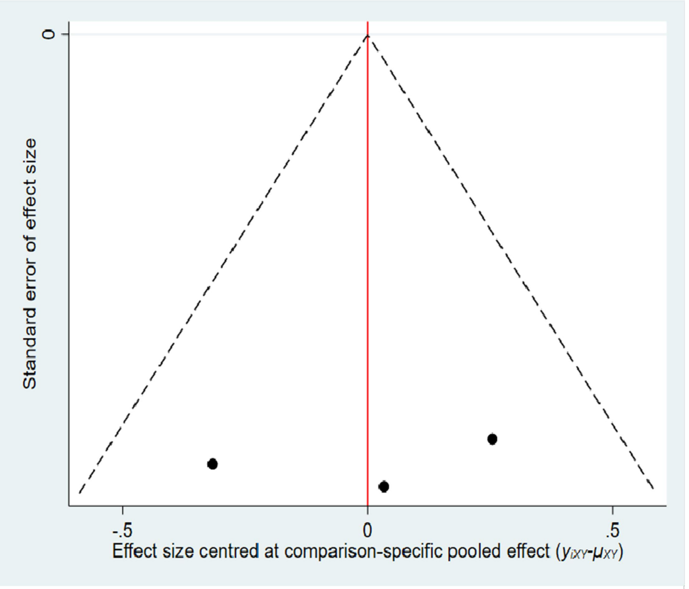

F. MNS ≥ 2 months

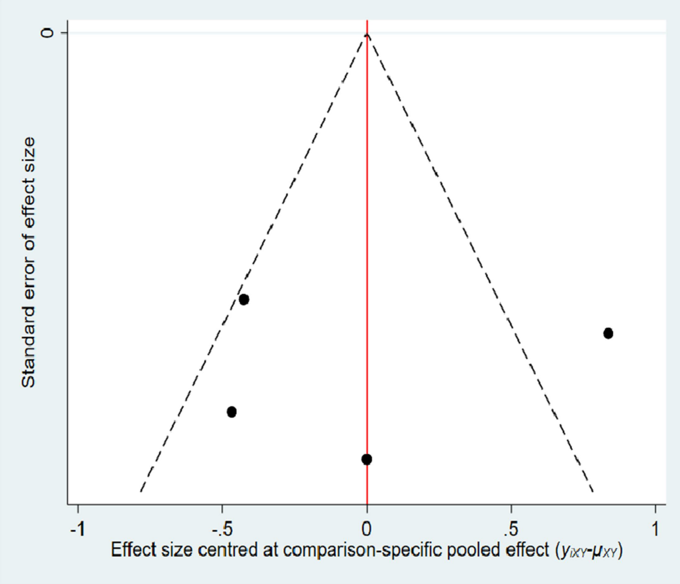

G. MGS < 2 months

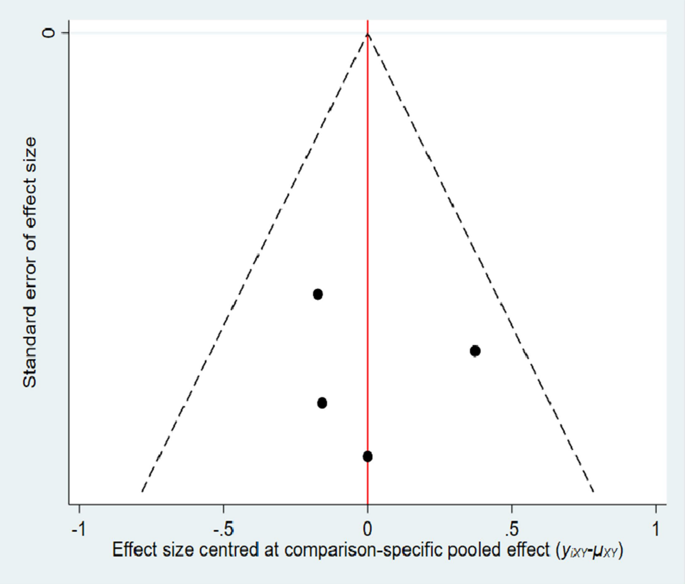

H. MGS ≥ 2 months

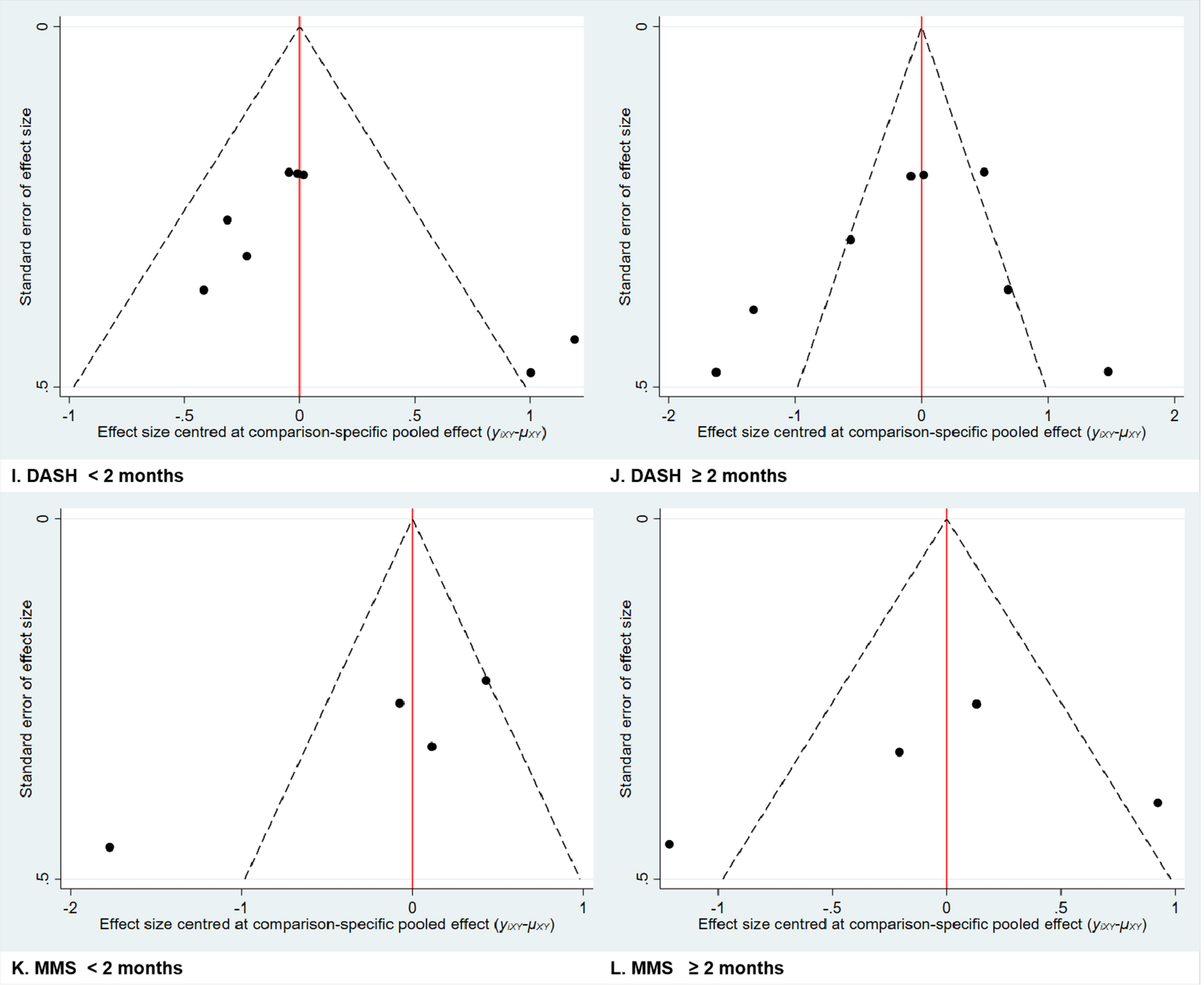
Standard error of effect size
Effect size centred at comparison-specific pooled effect (yiXY-μXY)
I. DASH < 2 months
J. DASH ≥ 2 months
K. MMS < 2 months
L. MMS ≥ 2 months

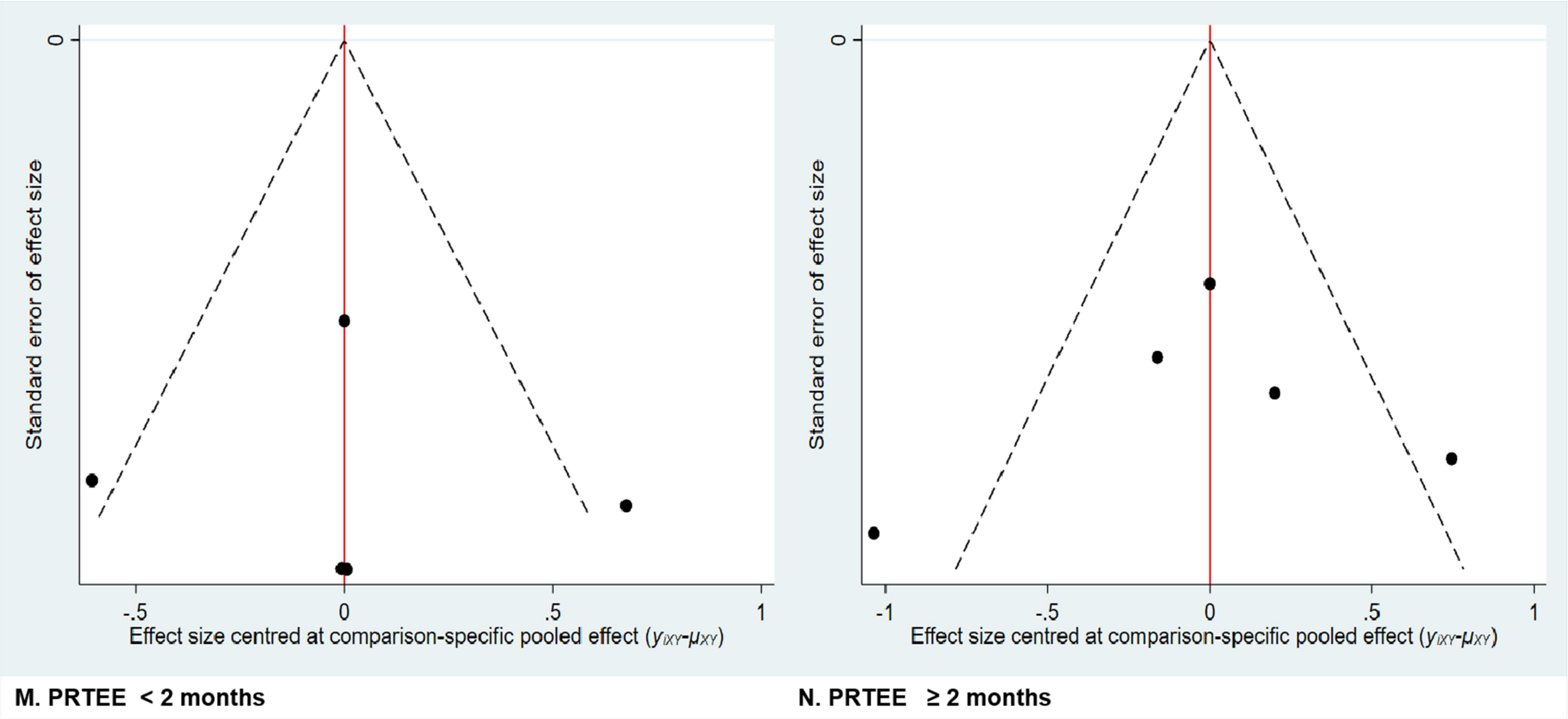

Supplement Fig. 4 The funnel plot is shown. VAS = visual analog score; MNS = modified Nirschl score; PPT = pressure pain threshold; MGS = maximum grip strength; DASH = Disabilities of the Arm Shoulder and Hand; MMS = modified Mayo score; PRTEE = Patient-Rated Tennis Elbow Evaluation.
